# Fasting induces a subcutaneous-to-visceral fat switch mediated by microRNA-149-3p and suppression of PRDM16

**DOI:** 10.1038/ncomms11533

**Published:** 2016-05-31

**Authors:** Hanying Ding, Shasha Zheng, Daniel Garcia-Ruiz, Dongxia Hou, Zhe Wei, Zhicong Liao, Limin Li, Yujing Zhang, Xiao Han, Ke Zen, Chen-Yu Zhang, Jing Li, Xiaohong Jiang

**Affiliations:** 1 State Key Laboratory of Pharmaceutical Biotechnology, Collaborative Innovation Center of Chemistry for Life Sciences, Jiangsu Engineering Research Center for MicroRNA Biology and Biotechnology, NJU Advanced Institute for Life Sciences (NAILS), School of life sciences, Nanjing University, 163 Xianlin Road, Nanjing, Jiangsu 210046, China; 2 Key Laboratory of Human Functional Genomics of Jiangsu Province, Nanjing Medical University, 140 Hanzhong Road, Nanjing 210029, China

## Abstract

Visceral adiposity is strongly associated with metabolic disease risk, whereas subcutaneous adiposity is comparatively benign. However, their relative physiological importance in energy homeostasis remains unclear. Here, we show that after 24-h fasting, the subcutaneous adipose tissue of mice acquires key properties of visceral fat. During this fast-induced ‘visceralization', upregulation of miR-149-3p directly targets PR domain containing 16 (PRDM16), a key coregulatory protein required for the ‘browning' of white fat. In cultured inguinal preadipocytes, overexpression of miR-149-3p promotes a visceral-like switch during cell differentiation. Mice deficient in miR-149-3p display an increase in whole-body energy expenditure, with enhanced thermogenesis of inguinal fat. However, a visceral-like adipose phenotype is observed in inguinal depots overexpressing miR-149-3p. These results indicate that in addition to the capacity of ‘browning' to defend against hypothermia during cold exposure, the subcutaneous adipose depot is also capable of ‘whitening' to preserve energy during fasting, presumably to maintain energy balance, via miR-149-3p-mediated regulation of PRDM16.

## Abstract (web summary)

Visceral adiposity is associated with metabolic diseases, whereas subcutaneous adiposity is comparatively benign. Here, the authors report that subcutaneous adipose tissue adopts visceral-like characteristics in response to prolonged fasting, and show this is mediated by miR-149-3p and its target, PRDM16.

## Graphical abstract

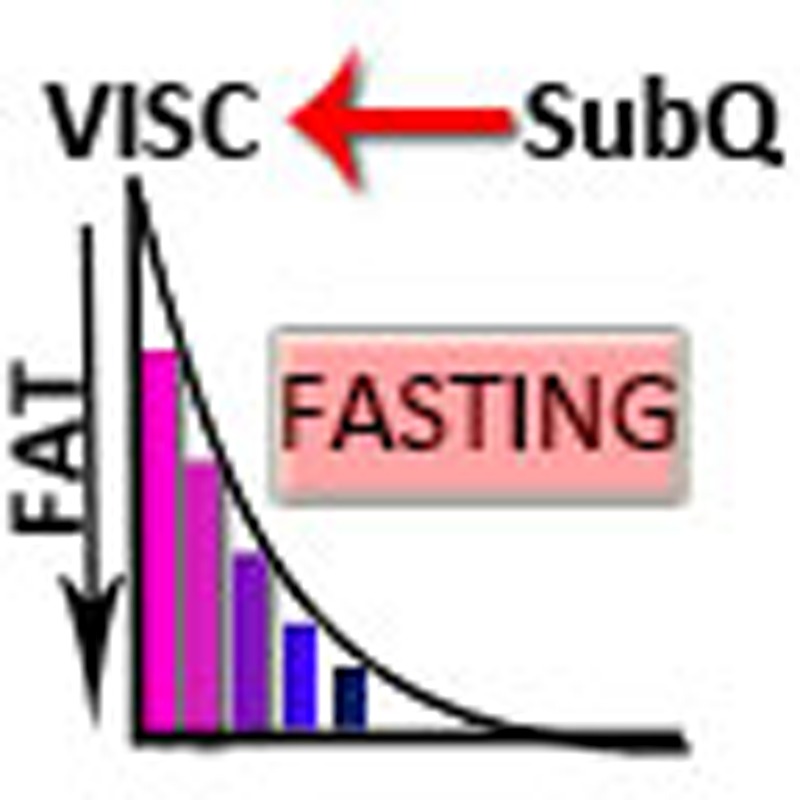

Adipocytes have been studied with increasing intensity due to the onset of the obesity epidemic. Traditionally, adipose tissues have been divided into two types: white adipose tissue (WAT), best known for storing excess energy in the form of triglycerides, and brown adipose tissue (BAT), which oxidizes chemical energy to produce heat to protect against hypothermia and obesity^1^,^2^,^3^. Most mammals have stereotypical adipose depots located throughout the body^4^. Classical BAT is typically located in the interscapular region in human infants and small mammals^5^. WAT generally develops in distinct intra-abdominal (visceral) depots and in the subcutaneous layer^1^. Scientists have recognized that the distribution of fat is closely linked to metabolic disease risk^6^. In particular, the accumulation of visceral WAT is strongly correlated with an increased risk of metabolic dysfunction and cardiovascular disease^7^,^8^,^9^. By contrast, the expansion of subcutaneous adiposity shows little or even an inverse correlation with disease risk^10^,^11^. Transplantation of subcutaneous depots, but not visceral fat, into the abdomen of mice leads to improved whole-body metabolism^12^,^13^. These divergent metabolic effects of different adipose depots have raised interest in the unique properties of visceral and subcutaneous fat^1^.

Recently, it has become clear that subcutaneous and visceral WAT have unique gene expression signatures^1^. Moreover, subcutaneous fat possesses substantial thermogenic capacity in response to cold stimulation compared with visceral depots^14^. A large accumulation of brown-like cells (termed beige/brite cells) during cold exposure is most prominent in the subcutaneous inguinal depot, whereas visceral adipocytes are less susceptible to ‘browning'^1^. The developmental and transcriptional control of beige cells have received much attention, mainly because of their potential roles in the defence against obesity and associated disorders.

As an integrator of energy homeostasis, many basic physiological functions provided by adipose tissue have been overlooked because of their association with obesity. Cold and hunger were probably the baseline states in humans over a substantial portion of evolutionary time^15^. Therefore, the lipid-burning brown/beige adipocytes are specialized to maintain body temperature by producing heat in a cold environment, whereas the lipid-storing white adipocytes are adapted to cope with food shortage^16^. The cold-induced emergence of brown-like adipocytes in subcutaneous WAT suggests that certain adipose processes are extraordinarily plastic in response to changes in environmental cues^17^,^18^. Fasting is defined as a coordinated set of metabolic changes that spare carbohydrate usage and increase reliance on fat as the energy supply^19^. Although several studies have reported that mobilization of the subcutaneous depot appears to be less than that of visceral fat during starvation, an important challenge is to understand the molecular mechanisms by which physiological changes regulate these different white adipose depots^20^.

PR domain containing 16 (PRDM16) is a zinc-finger protein that functions as a bidirectional cell fate switch between skeletal myoblasts and promotes BAT differentiation^2^,^3^. In addition, ectopic expression of PRDM16 in adipocytes strongly induces the thermogenic programme in subcutaneous depots but not in visceral fat^5^. Notably, PRDM16 mRNA and protein levels are depot dependent, likely due to differential stability of the PRDM16 protein in subcutaneous and visceral depots^1^,^20^. Significant attention has been focused on the role of microRNAs (miRNAs) in adipocyte function. In particular, several miRNAs have a preferential effect on brown and beige cell differentiation and function, including some that target PRDM16 (refs 21, 22, 23, 24, 25).

Here, we report that, after 24 h of food deprivation, subcutaneous inguinal WAT (ingWAT) takes on many of the morphological and molecular characteristics of visceral fat to preserve energy via miR-149-3p-mediated suppression of PRDM16. These data unravel the critical role of subcutaneous WAT in regulating the energy balance through miRNA-mediated regulation of PRDM16.

## Results

### Fasting mobilizes more visceral fat than subcutaneous depot

**Figure 1:**
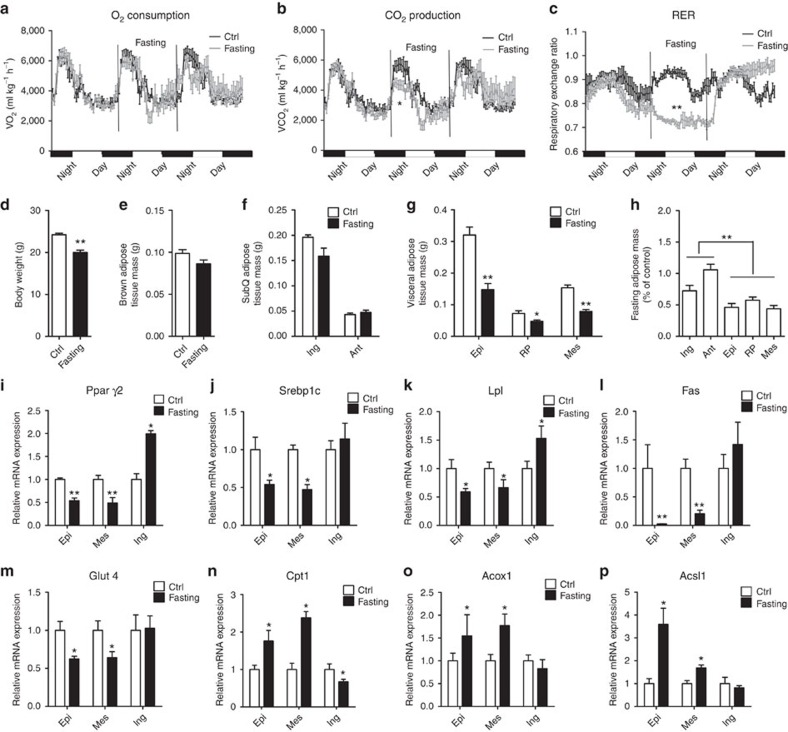
Fasting induces depot-specific mobilization of lipids in different adipose tissues. (**a**–**c**) O_2_ consumption (**a**), CO_2_ production rates (**b**) and the respiratory exchange ratio (RER) (**c**) were measured by indirect calorimetry from ad libitum fed or 24 h-fasted male mice (*n*=6). (**d**–**h**) Male mice (*n*=8) were fed ad libitum or fasted for 24 h (*n*=8). Body weight (**d**), weight of brown fat mass (**e**), subcutaneous fat mass (**f**), visceral fat mass (**g**). Decreased ratios of subcutaneous and visceral fat mass (**h**). (**i**–**p**) Expression of lipogenic and lipolytic genes in epididymal, mesenteric and inguinal depots from ad libitum fed or 24 h-fasted mice (*n*=8). The same amount of RNA was used for reverse transcription followed by real-time PCR. Gene expression was normalized to housekeeping gene *Gapdh*. Relative levels of the lipogenic genes *Pparγ2* (**i**), *Srebp1c* (**j**), *Lpl* (**k**), *Fas* (**l**), *Glut4* (**m**), and the lipolytic genes *Cpt1* (**n**), *Acox1* (**o**), *Acsl1* (**p**) are shown (*n*=8). Ant, anterior; Epi, epididymal; Ing, inguinal; Mes, mesenteric; RER, respiratory exchange ratio; RP, -retroperitoneal; SubQ, subcutaneous. The data represent the mean±s.e.m. *P<0.05; **P<0.001; (Student's *t*-test).

The early stages of fasting last for up to 24 h as the body adjusts to the absence of nutrient ingestion^19^. Therefore, we tested the relevant metabolic parameters in mice fed *ad libitum* or fasted for 24 h. Decreased CO_2_ production resulted in a markedly reduced respiratory exchange ratio (RER) in male mice, indicating that 24-h fasting stimulated a substantial shift from carbohydrate to fat-based fuel (Fig. 1a–c). Fasting for 24 h significantly reduced the body weight of both male and female mice (Fig. 1d; Table 1). Six adipose depots, including interscapular BAT (BAT); three representative visceral WATs, -retroperitoneal (-RP), -mesenteric (--mes) and epididymal (-epi, male)/periovarian (-PO, female) WAT; and two major subcutaneous WATs, -inguinal (-ing) and anterior (-Ant) WAT, were collected from male and female mice. Although a trend towards decreased weight was observed in the examined male and female depots, the weight of the BAT was mainly unchanged in fasted mice (Fig. 1e; Table 1). Compared with the subcutaneous depot, which exhibited a moderate reduction in weight, the weight of the visceral depots was significantly decreased in both male and female mice. Gonadal fat (-Epi for males, -PO for females) was decreased by ∼50% in mice, and greater than 50% fat loss was observed in the mesenteric depot, which also exists in larger mammals include humans (Fig. 1f–h; Table 1). These results indicate that the mobilization of subcutaneous and visceral fat pads during fasting was different in male and female mice. To investigate possible depot-specific responses in fasted mice, lipogenic-related genes were measured. The mRNA levels of *Pparγ2* (peroxisome proliferator-activated receptor γ2) and *Srebp1c* (sterol regulatory element binding protein 1c), two main lipogenic transcription factors, were significantly decreased in the epiWAT and mesWAT of fasted mice. Strikingly, twofold upregulation of *Pparγ2* and a slight increase in *Srebp1c* mRNA levels were observed in the ingWAT of fasted mice (Fig. 1i,j). Fasting also decreased the mRNA levels of fatty acid synthesis-related genes in the epiWAT and mesWAT, including *Lpl* (lipoprotein lipase), *Fas* (fatty acid synthesis) and *Glut4* (glucose transporter type 4); however, these genes were not significantly altered in the ingWAT (Fig. 1k–m). Moreover, the mRNA levels of *Cpt1* (carnitine palmitoyltransferase 1), *Acox1* (acyl-coenzyme A oxidase 1) and *Acsl1* (Acyl-CoA synthetase long-chain 1), three lipolysis genes involved in fatty acid oxidation, were markedly increased in fasted epiWAT and mesWAT, whereas the same set of genes showed a tendency towards a reduction in ingWAT (Fig. 1n-p). Similar depot-specific responses were observed in female fasted mice (Supplementary Fig. 1a–h). These results suggest that 24-h fasting triggered differential responses among adipose tissues in male and female mice. Specifically, the preferential mobilization of lipids in response to fasting was observed in the more internally located visceral WAT compared with the relatively externally located subcutaneous ingWAT.

### Fasting drives visceral-like phenotype switches in ingWAT

**Figure 2:**
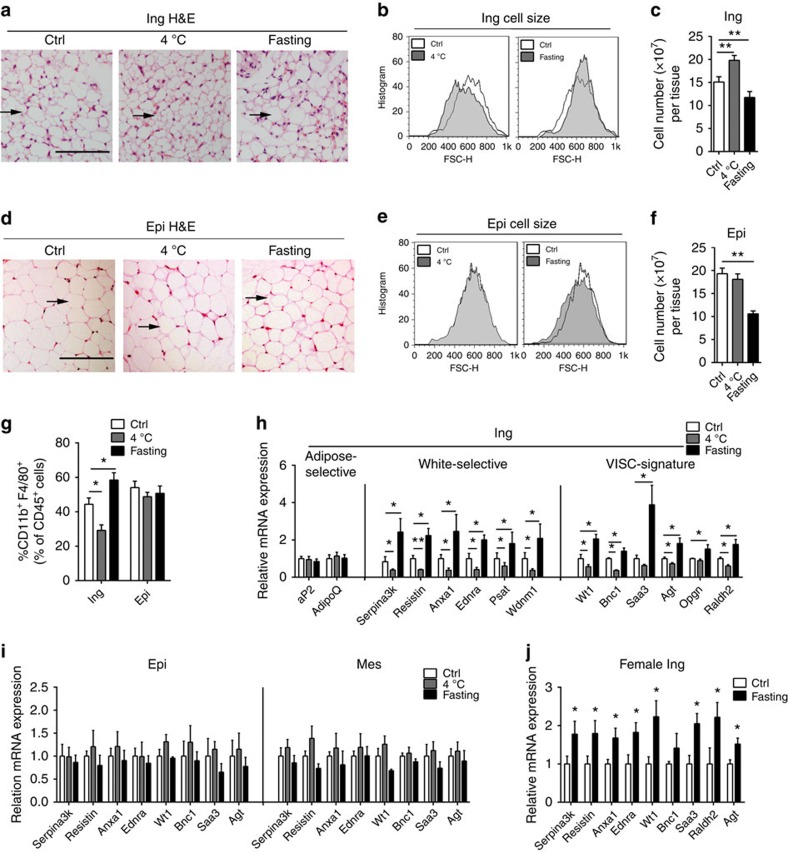
Fasting drives visceral-like morphological and molecular phenotypes in ingWAT. (**a**) Representative images from haematoxylin and eosin (H&E) stained sections of inguinal adipose tissue. Scale bar, 100 μm. (**b**) Histograms showing cell size (FSC-) in inguinal adipose tissue from ad libitum fed (2 × 10^4^ cells per sample), 24 h-fasted or 24 h cold-exposed (4 °C) male mice (*n*=8). (**c**) Absolute quantification of cell number in inguinal adipose tissue from the three groups of male mice (*n*=8). (**d**) Representative images from haematoxylin and eosin (H&E) stained sections of epididymal adipose tissue. Scale bar, 100 μm. (**e**) Histograms show cell size (FSC-) in epididymal adipose tissue from ad libitum fed (2 × 10^4^ cells per sample), 24-h fasted or 24-h cold-exposed (4 °C) male mice (*n*=8). (**f**) Absolute quantification of cell number in epididymal adipose tissue from the 3 groups of male mice (*n*=8). (**g**) Flow cytometric quantitation of CD11b^+^F4/80^+^ macrophages in inguinal, epididymal and mesenteric adipose tissue from ad libitum fed, 24-h fasted mice or 24-h cold exposed (4 °C) male mice (*n*=6). (**h**) Normalized expression of general adipose marker genes, white fat-selective genes, and visceral signature genes in inguinal adipose tissue from ad libitum fed, 24 h-fasted or 24 h-cold-exposed (4 °C) male mice (*n*=8). (**i**) Normalized expression of white-selective and visceral signature genes in epididymal and mesenteric adipose tissue from the three groups of male mice (*n*=8). (**j**) Normalized expression of white-selective and visceral signature genes in inguinal adipose tissues from *ad libitum*-fed or 24 h-fasted female mice (*n*=8). Epi, epididymal; Ing, inguinal; Mes, mesenteric; VISC, visceral. The data present the mean±s.e.m. *P<0.05; **P<0.001; (Student's *t*-test).

We next tested whether 24-h fasting also influenced the morphologies of different adipose tissues. A small proportion of ‘multilocular' brown-fat-like cells mixed with regions of ‘unilocular' fat cells were readily observed in the subcutaneous ingWAT of male C57BL/6J mice that were maintained at ambient temperature (25 °C). Therefore, we examined the effects of two potent fat stimuli in mice: 24-h cold exposure (4 °C)—a physiological stimulator of subcutaneous fat ‘browning' and 24-h fasting. Cold exposure resulted in increased clusters of multilocular brown fat-like areas and decreased unilocular white regions in the ingWAT. However, fasting led to a significant decrease in multilocular brown fat-like areas along with increased unilocular white regions, suggesting that a visceral-like switch might occur in ingWAT (Fig. 2a). Although the 24-h fast did not influence the morphology of BAT (multilocular) or epiWAT (unilocular), a tendency towards reduced cell size was observed in the fasted epiWAT (Fig. 2d; Supplementary Fig. 2a). Next, we used flow cytometry to measure the changes in cell size and number in ingWAT/epiWAT after a 24-h fast. For the ingWAT, cold exposure induced an increase in the proportion of smaller adipocytes (FSC-H shift to the left). Conversely, a sharp decrease in smaller adipocytes was observed in the fasted ingWAT (FSC-H shift to the right); however, the size of the unilocular adipocytes was relatively stable (Fig. 2b). The ‘vanishing' of the smaller adipocytes might account for the moderate weight loss observed for the fasted ingWAT, which resulted in decreased adipocyte numbers along with an increased proportion of unilocular adipocytes in ingWAT (Fig. 2c). For epiWAT, 24 h of cold exposure yielded no significant alteration of adipocyte size or number. However, the histogram showed that 24-h fasting caused a moderate decrease in epididymal cell size, which is consistent with the histological analysis (Fig. 2d,e). Moreover, the number of epididymal adipocytes was markedly decreased (Fig. 2f). Therefore, the decreased cell volume and the significantly decreased cell number presumably account for the marked weight loss in the epiWAT. We also quantified the major myeloid and lymphoid subsets in the ingWAT of different groups of mice^26^. Compared with control animals, ingWAT from cold-exposed mice exhibited a 35% decrease in the number of CD11b^+^F4/80^+^ macrophages, whereas ingWAT from fasted mice exhibited a 30% increase (Fig. 2g). These alterations were restricted to subcutaneous ingWAT because no differences were observed in visceral epiWAT or BAT (Fig. 2g; Supplementary Fig. 2b). These data confirm previous observations of a visceral-like phenotype in the fasted ingWAT. Thus, two sets of marker genes were analysed in ingWAT and epiWAT from cold-exposed mice and fasted mice, including classical WAT-selective genes and newly identified visceral signature genes by Cohen *et al*.^26^. The classic WAT-selective genes, including *Serpina3k* (serine peptidase inhibitor, clade A, member 3k), *Resistin*, *Anxa1* (annexin A1), *Endra* (endothelin receptor type A), *Psat* (phosphoserine aminotransferase) and *Wdnm1* (WDNM1-like protein), were markedly increased in the fasted ingWAT. Moreover, the newly identified visceral signature genes^3^,^26^, which include two transcription factors (*Wt1* (Wilms tumour 1) and *Bnc1* (basonuclin 1)) and several proinflammatory genes (*Saa3* (serum amyloid A3)*, Agt* (angiotensinogen)*, Opgn* (osteoprotegerin) and *Raldh2* (retinaldehyde dehydrogenase 2)) were also significantly upregulated in the fasted ingWAT. However, both sets of genes were expressed at reduced levels in the cold-exposed ingWAT. Neither cold exposure nor fasting influenced the expression of the general adipogenic markers *aP2* (adipocyte protein 2) and *AdipoQ* (adiponectin C1Q and collagen domain containing) (Fig. 2h). These results confirmed that a visceral-like switch occurred at the molecular level in the fasted ingWAT. We also tested these representative WAT/visceral-selective genes in BAT and visceral WAT (epiWAT and mesWAT). Neither the classic WAT-selective nor the visceral signature genes were significantly altered in these adipose tissues (Fig. 2i; Supplementary Fig. 2c). These results were also confirmed in female mice, indicating that fasting can drive a subcutaneous-to-visceral-like switch at both the morphological and molecular levels (Fig. 2j; Supplementary Fig. 2d).

### Fasting suppresses ingWAT thermogenesis by inhibiting PRDM16

**Figure 3:**
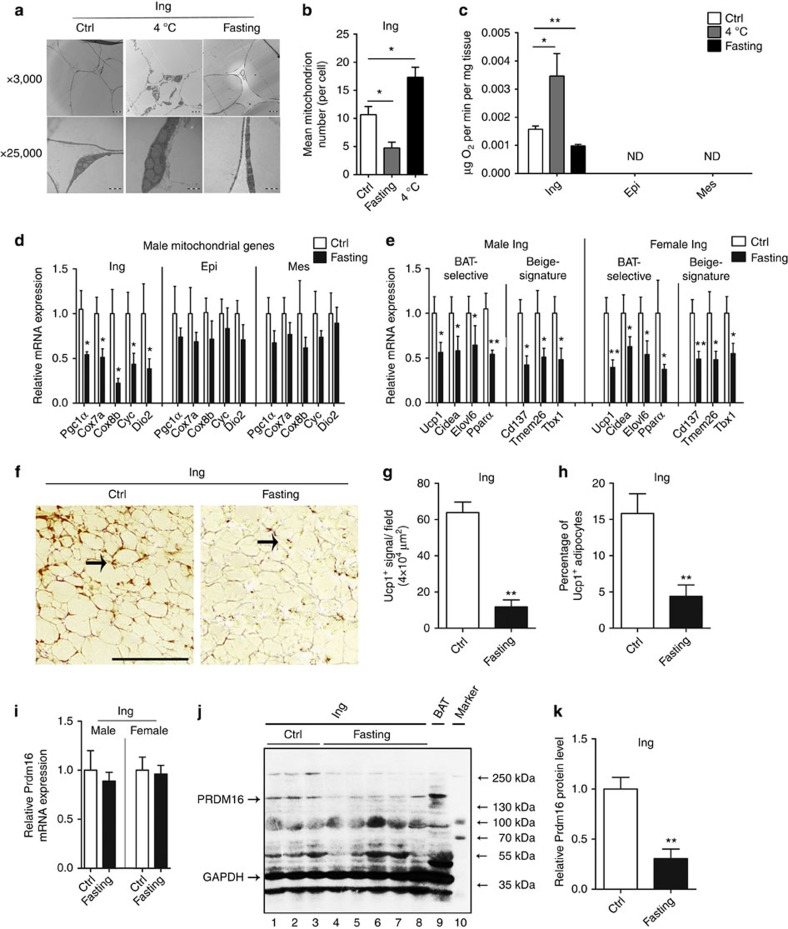
Fasting induces a functional visceral-like switch in ingWAT. (**a**) Transmission electron microscopy of inguinal adipose tissue in the three groups of mice. Scale bar, 5μm. (**b**) Mitochondrial numbers in inguinal adipose tissue sections from male mice (*n*=5). (**c**) O_2_ consumption in inguinal, epididymal and mesenteric white adipose tissue from the three groups of mice (*n*=6). (**d**) Normalized expression of mitochondrial component genes in inguinal, epididymal and mesenteric adipose tissue in fasted mice compared with that in male control mice (*n*=8). (**e**) Normalized thermogenic genes in inguinal adipose tissues from the ad libitum and 24 h-fasted male and female mice (*n*=8). (**f**–**h**) Quantification of UCP1 protein in inguinal subcutaneous adipose tissue from *ad libitum*-fed and, 24-h-fasted male mice. (**f**) Representative images of sections from UCP1 immunohistochemistry. Scale bar, 100 μm. (**g**) Quantification of UCP1^+^ signals per field (**h**), and percentage of UCP1^+^ cells in sections (*n*=5). (**i**–**k**) Analysis of Prdm16 mRNA (*n*=8) (**i**) and protein levels (**j**,**k**) in the inguinal adipose depots from ad libitum fed and 24 h-fasted male mice. GAPDH was used as an internal control. BAT; brown adipose tissue; Epi, epididymal; Ing, inguinal; Mes, mesenteric. The data represent the mean±s.e.m. *P<0.05; **P<0.001; (Student's *t*-test).

A defining feature of subcutaneous ingWAT is its relatively abundant mitochondria and associated higher capacity for thermogenesis compared with visceral WAT (Supplementary Fig. 3a). Representative electron micrographs showed that cold exposure elevated the number of mitochondria in the ingWAT of mice, whereas fasting resulted in a marked decrease in the number of mitochondria (Fig. 3a,b). We next used O_2_ consumption as a readout to assess the physiological effects of cold exposure and fasting on adipose tissue. The O_2_ consumption of the ingWAT increased twofold in cold-exposed mice but decreased about 50% in fasted mice compared with control mice. The O_2_ consumption in the epiWAT and mesWAT was below the limit of detection, and no significant alteration was observed in BAT (Fig. 3c; Supplementary Fig. 3b). The mRNA levels of mitochondrial oxidation associated genes (*Pgc-1α* (peroxisome proliferator-activated receptor gamma, coactivator 1α), *Cox7a* (cytochrome c oxidase subunit 7a), *Cox8b* (cytochrome c oxidase subunit 8b), *Cyc* (cytochrome c) and *Dio2* (type II iodothyronine deiodinase)), BAT-selective genes (*Ucp1*(uncoupling protein 1), *Cidea* (cell death-inducing DNA fragmentation factor, alpha subunit-like effector a), *Elovl6* (elovl fatty acid elongase 6) and *Pparα* (peroxisome proliferator activator receptorα) and beige-signature genes (*Cd137*(tumour necrosis factor receptor superfamily, member 9), *Tmem26* (transmembrane protein 26) and *Tbx1* (T-box 1)), which are closely related to the thermogenic capacity, were profoundly decreased in the fasted ingWAT, suggesting that a significant functional visceral-like switch occurred in the fasted ingWAT (Fig. 3d,e). Considering that the whole-body switches to a thrifty mode to reduce energy expenditure during fasting, mitochondrial and BAT-selective genes were also slightly decreased in both BAT and visceral WAT (epiWAT/mesWAT) (Fig. 3d; Supplementary Fig. 3c). Immunohistochemical analysis revealed that a certain amount of UCP1 was readily observed in the ingWAT of control mice, whereas UCP1 was almost undetectable in the fasted ingWAT (Fig. 3f–h). Because cold exposure is clearly different from fasting, we also performed similar sets of experiments on mice exposed to cold combined with fasting for 24 h. When mice were exposed to cold, the 24-h fast did not efficiently induce morphological, molecular or functional ‘visceralization' of ingWAT, indicating that cold diminished the effect of fasting on visceral switching (Supplementary Fig. 3d–h). Emerging evidence suggests fascinating effects of intermittent fasting^27^. Therefore, we performed intermittent fasting on mice, by alternating 24-h cycles of fasting and *ad libitum* feeding. The visceral fats decreased, which might account for the observation of a slight decrease in body weight (Supplementary Fig. 3i,k). This alternate day-fasting (ADF) also markedly increased the expressions of mitochondrial biogenesis genes in the epiWAT of the mice (Supplementary Fig. 3l). Notably, unlike visceral fat, both the decreased weight and the impaired mitochondrial biogenesis were restored in subcutaneous depots by 24-h refeeding, suggesting that the ‘visceralization' of the ingWAT is an adaptive response to 24-h fasting-induced physiological stress to maintain whole-body energy homeostasis (Supplementary Fig. 3j,l). These results confirmed that ADF preferentially consumes the ‘metabolically harmful' visceral fat, and that long-term ADF might benefit health^28^,^29^,^30^. Intriguingly, the 24-h fast did not significantly influence on the mRNA level of *Prdm16* (Fig. 3i). Strikingly, the protein level of PRDM16 decreased markedly in the fasted ingWAT of both male and female mice (Fig. 3j,k; Supplementary Fig. 3m,n). Because PRDM16 is a critical mediator of adaptive thermogenesis in subcutaneous WAT, fasting might suppress the thermogenic programme in the ingWAT mainly by suppressing the protein level of PRDM16. The inconsistency in mRNA and protein levels strongly suggests that a post-transcriptional mechanism may function in the regulation of PRDM16. Because the expression levels of *Gapdh* (glyceraldehyde-3-phosphate dehydrogenase) and *36b4* were stable in both fasted and cold-exposed mouse samples (Supplementary Fig. 3o), the relative gene expression levels were obtained by normalization to *Gapdh* and confirmed by *36b4* (Supplementary Fig. 3p).

### miR-149-3p directly targeting PRDM16 in subcutaneous ingWAT

**Figure 4:**
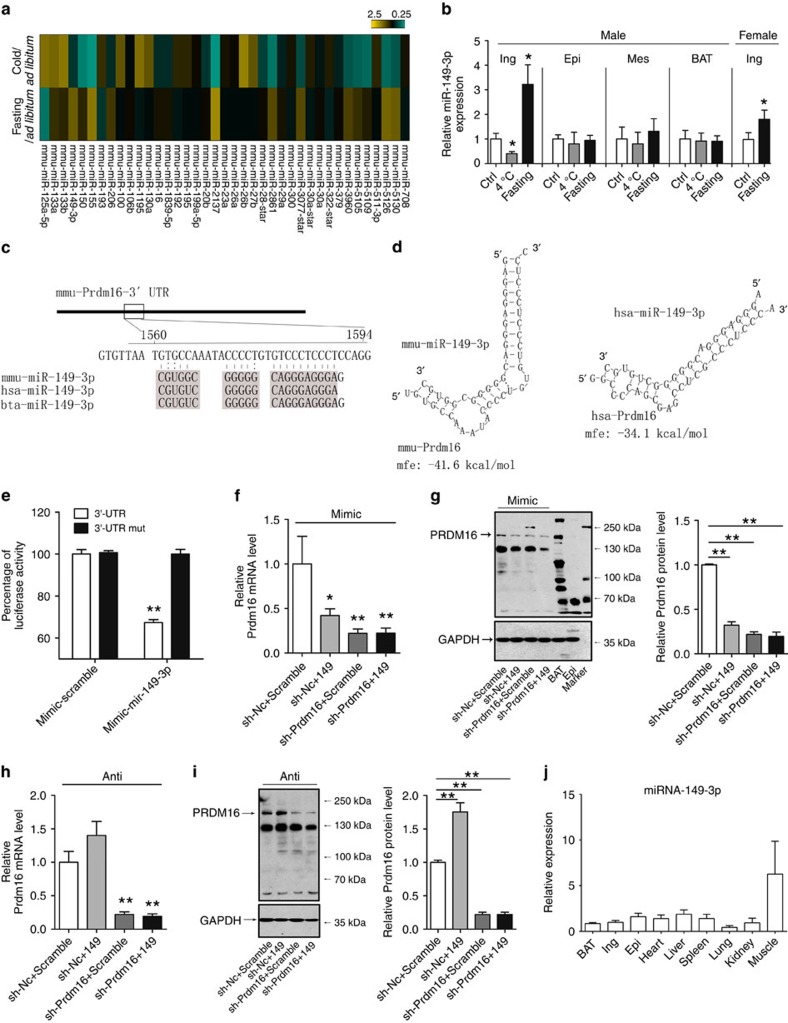
Prdm16 is directly targeted by miR-149-3p. (**a**) Heat map showing the relative expression of miRNAs that changed in the opposite direction in inguinal adipose tissues of fasted and cold exposed mice. Each sample comprised a pool of inguinal adipose tissues from four animals. Each column depicts an individual miRNA. Each row depicts the miRNA expression in fasted or cold exposed samples relative to the expression in control mice. The fold change for the samples is colour-coded according to the key. (**b**) Relative expression level of miRNA-149-3p normalized to snRNAU6 measured by quantitative real-time PCR in inguinal, epididymal, mesenteric and brown adipose tissue from cold-exposed and fasted male mice, or in inguinal adipose tissue from fasted female mice (*n*=8). (**c**) Putative miRNA target sites of miR-149-3p within the 3′-UTR of Prdm16. (**d**) Bioinformatic prediction of miR-149-3p target sites and free energy values within the 3′-UTRs of mouse and human Prdm16. (**e**) Relative luciferase activity in HEK293T cells transfected with plasmid reporter constructs containing the 3′-UTR or mutated 3′-UTR of Prdm16, co-transfected with mimic-miR-149-3p (*n*=6). (**f**,**g**) Prdm16 mRNA (**f**) and protein (**g**) levels in two-day differentiated inguinal SV cells infected with adenovirus expressing a shRNA targeted to Prdm16 or a scrambled control shRNA (sh-Nc), co-transfected with scramble or miR-149-3p mimic. (**h**,**i**) PRDM16 mRNA (**h**) and protein (**i**) levels in two-day differentiated inguinal SV cells infected with adenovirus expressing a shRNA targeted to Prdm16 or a scrambled control shRNA (sh-Nc), co-transfected with scramble or miR-149-3p anti-miRs. (**j**) Relative miR-149-3p expression level in different tissues of mice measured by RT–PCR (*n*=8). BAT; brown adipose tissue; Epi, epididymal; Ing, inguinal; Mes, mesenteric. The data represent the mean±s.e.m. *P<0.05; **P<0.001; (Student's *t*-test).

To investigate whether miRNAs are involved in the regulation of PRDM16 in the ingWAT in response to different physiological stimuli, we performed miRNA microarray analysis using ingWATs from the cold-exposed, -fasted and control mice. Given that cold exposure increased the protein level of PRDM16 whereas fasting decreased its expression, miRNAs with expression patterns opposite that of PRDM16 were selected (Fig. 4a; Supplementary Fig. 4a,b). Using two computational algorithms TargetScan and miRanda, miR-149-3p, which has a conserved target site with the seed sequence in the 3′UTR of the Prdm16 mRNA, was selected for further experimental verification. The conservation of the seed sequence suggests biological relevance for these miRNAs in the regulation of Prdm16 expression in humans (Fig. 4c,d). Quantitative RT–PCR (PCR with reverse transcription) assays verified that cold exposure significantly decreased miR-149-3p expression, whereas fasting resulted in a marked increase in miR-149-3p in both male and female mice. Of note, neither cold nor fasting markedly changed the expression of miR-149-3p in BAT or visceral (-epi, -mes) WAT (Fig. 4b). However, when mice were exposed to cold, fasting failed to induce miR-149-3p expression in ingWAT, and it subsequently led to a relatively stable level of PRDM16 protein (Supplementary Fig. 4a,b). Notably, the expression of reported myomiR-133 was also analysed by quantitative RT–PCR analysis. We confirmed that cold exposure decreased the level of miR-133a in ingWAT^21^, whereas fasting induced its expression, suggesting that miR-133a might also play a role in ingWAT in response to changes in physiological conditions change (Supplementary Fig. 4c). Because myomiR-133 has been reported to regulate brown fat differentiation through Prdm16, we focused on the role of the newly identified candidate miR-149-3p in the following study. Moreover, another miRNA cluster, miR-193b/365 (ref. 25), which has been reported to be regulated by PRDM16 in classical brown fat, was not significantly altered in BAT, ingWAT or epiWAT when the mice were exposed to cold or fasting (Supplementary Fig. 4d,e). We next performed luciferase assays to investigate the direct targeting of the Prdm16 3′-UTR by miR-149-3p. Human embryonic kidney 293 T (HEK293T) cells transfected with reporter plasmids containing the Prdm16 3′-UTR showed markedly decreased luciferase activity in the presence of ectopic miR-149-3p. Mutation of the conserved seed sequence abrogated the miRNA-induced repression of the Prdm16 3′-UTR (Fig. 4e). We also knocked down the expression of Prdm16 in primary cultured ingWAT stromal-vascular (SV) cells using a shRNA expressed from an adenovirus. Adenoviral vectors expressing a control scrambled sequence or Prdm16 shRNA (sh-Prdm16) were used to infect sub-confluent cultured SV cells from ingWAT, and these cells were transfected with miR-149-3p mimic when induced to undergo adipogenesis 2 days after adenovirus transduction. After 2 days of differentiation, transfer of the miR-149-3p mimic resulted in a significant reduction of the PRDM16 protein level in control (scrambled) cells along with decreased *Prdm16* mRNA expression (Fig. 4f,g; Supplementary Fig. 4f). Conversely, inhibition of the miRNA using an anti-miR-149-3p oligonucleotide markedly increased the protein level of PRDM16 in control cells. The mRNA level of Prdm16 was largely unchanged in cells transfected with the anti-miR-149-3p, although a trend towards elevation was observed (Fig. 4h,i; Supplementary Fig. 4g). However, because the levels of PRDM16 mRNA and protein were both very efficiently decreased (greater than 75% reduction) by the sh-Prdm16 vectors in adipocytes, transfection with neither the miR-149-3p mimic nor the anti-miR-149-3p oligonucleotide significantly altered the PRDM16 protein level (Fig. 4g,i). These data suggest that PRDM16 is a direct target of miR-149-3p in subcutaneous ingWAT. As shown in Fig. 4j, because miR-149-3p has rarely been reported, we also measured its expression level in different mouse tissues.

### Inhibition of miR-149-3p stimulates Ing adipocytes browning

**Figure 5:**
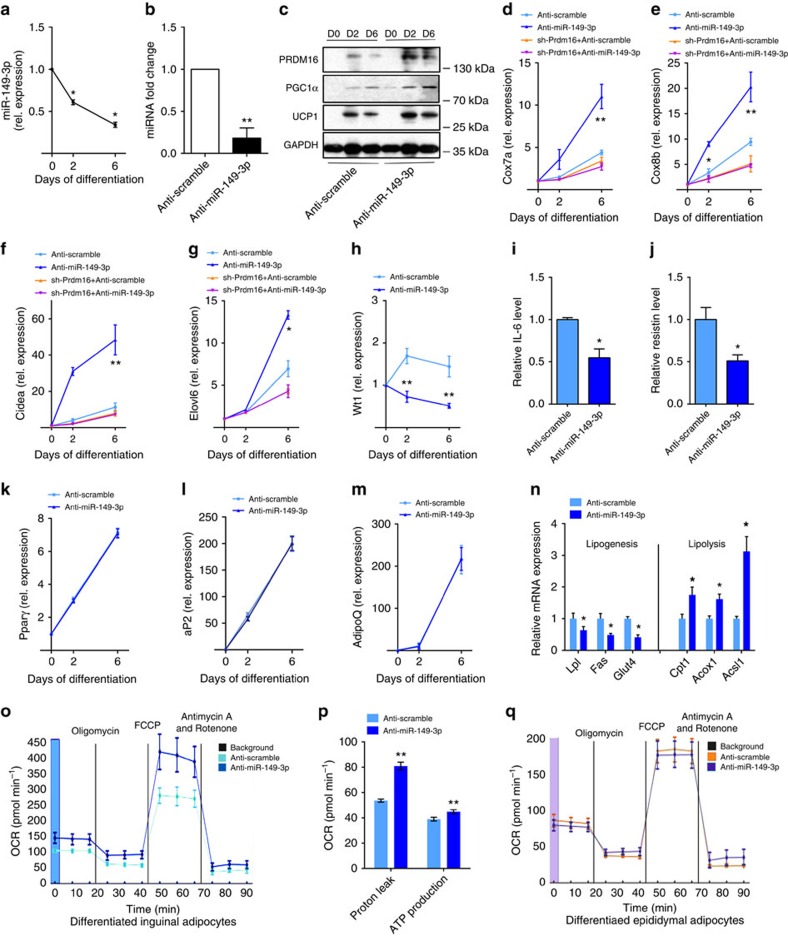
Inhibition of miR-149-3p induces thermogenesis in differentiated inguinal adipocytes. (**a**) Relative miR-149-3p expression during primary inguinal SV differentiation using an induction cocktail, measured by quantitative real-time PCR with normalization to U6. (**b**) Relative expression level of miR-149-3p in inguinal SV cells transfected with anti-miR-149-3p. (**c**) Western blot analysis of PRDM16, PGC-1α and UCP1 levels in inguinal SV cells transfected with anti-miR-149-3p or anti-miR-control (anti-scramble), at the indicated time points (day 0, day 2 and day 6) during inguinal SV cell differentiation. (**d**–**g**) Relative mRNA expression of *Cox7a* (**d**), *Cox8b* (**e**), *Cidea* (**f**), and *Elovl6* (**g**) in inguinal SV cells infected with adenovirus expressing a shRNA targeted to Prdm16 co-transfected with anti-miR-149-3p or anti-miR-control during differentiation. (**h**) Relative mRNA expression of Wt1. (**i**,**j**) ELISA analysis of IL-6 (**i**) and Resistin (**j**) expression in inguinal SV cells in differentiation medium at day 6. (**k**–**m**) Relative mRNA expression of the adipogenic marker genes *Pparγ* (**k**), *aP2* (**l**) and *AdipoQ* (**m**) in inguinal SV cells transfected with anti-miR-149-3p or anti-miR-control during differentiation. (**n**) Relative mRNA expression of lipogenesis and lipolysis genes in inguinal SV cells at day 6. The data show the mean of five independent experiments. (**o**–**q**) Oxygen consumption rates (OCRs) were quantified under basal conditions and with drugs that disrupt the respiratory chain using a Seahorse Biosciences XF 96 analyser in 6-day differentiated inguinal adipocytes (**o**,**p**) or epididymal adipocytes (**q**) transfected with anti-miR-149-3p or anti-miR-control. Experiments were performed in triplicated wells for each condition and repeated five times independently. The data present the mean±s.e.m. *P<0.05; **P<0.001; (Student's *t*-test).

To identify whether miR-149-3p alters the function of subcutaneous adipocytes, we isolated SV cells from the ingWAT of mice and induced their differentiation into beige adipocytes (Supplementary Fig. 5a). miR-149-3p was significantly downregulated during differentiation (Fig. 5a). To mimic physiological conditions, we used a relatively low dose of anti-miR to inhibit miRNA expression (Fig. 5b). The inhibition of miR-149-3p increased the protein level of PRDM16 approximately sixfold at day six of differentiation, compared with an approximately threefold increase in cells treated with scrambled anti-miR. miR-149-3p inhibition also caused significant increases in PGC-1α and UCP1 protein levels compared with controls (Fig. 5c; Supplementary Fig. 5b). Next, to examine whether miR-149-3p alters the function of inguinal adipocytes to dissipate energy in a PRDM16-dependent manner, thermogenic genes in control and PRDM16-deficient cells were measured in the presence or absence of miR-149-3p. Inhibition of miR-149-3p markedly increased the set of brown-selective genes *Cox7a, Cox8b, Cidea* and *Evovl6*. However, in PRDM16-deficient cells, these genes were not altered in the absence of miR-149-3p, suggesting that miR-149-3p acts through PRDM16 (Fig. 5d–g). Conversely, inhibition of miR-149-3p decreased the mRNA level of the visceral-selective marker *Wt1* in adipocytes, and it subsequently suppressed IL-6 and Resistin, two representative WAT-selective secreted proteins in culture medium (Fig. 5h–j). Notably, miR-149-3p inhibition did not affect inguinal adipocyte differentiation *per se* (Supplementary Fig. 5c). The expression levels of three genes common to both white and brown fat cells, *Pparγ, aP2* and *AdipoQ*, were similar in the presence or absence of miR-149-3p (Fig. 5k-m). Moreover, at day six of differentiation, the mRNA levels of the fatty acid synthesis-related genes *Lpl*, *Fas* and *Glut4* were significantly repressed, whereas *Cpt1a, Acox1* and *Acsl1*, three genes involved in fatty acid oxidation, were markedly increased by miR-149-3p inhibition (Fig. 5n). To further address the functional properties, we performed real-time bioenergetic kinetics on differentiated inguinal adipocytes. A higher oxygen consumption rate (OCR) from proton leakage and an increase in the maximal respiratory capacity were observed in adipocytes after miR-149-3p inhibition (Fig. 5o,p). These data demonstrate that depletion of miR-149-3p during inguinal adipocyte differentiation increased mitochondrial activity levels, which is an important functional characteristic of BAT. Because miR-149-3p is also expressed in visceral epiWAT, to examine whether miR-149-3p plays a role in visceral fat cells, we isolated SV cells from the epiWAT of mice and performed the same set of experiments. However, inhibition of miR-149-3p did not alter the thermogenic programme, lipogenesis/lipolysis or mitochondrial respiration of epididymal adipocytes, suggesting that miR-149-3p might have tissue specific roles (Fig. 5q; Supplementary Fig. 5d–o). This is reasonable considering that PRDM16 expression is much lower in epiWAT compared with ingWAT.

### miR-149-3p induces Ing adipocytes visceral differentiation

**Figure 6:**
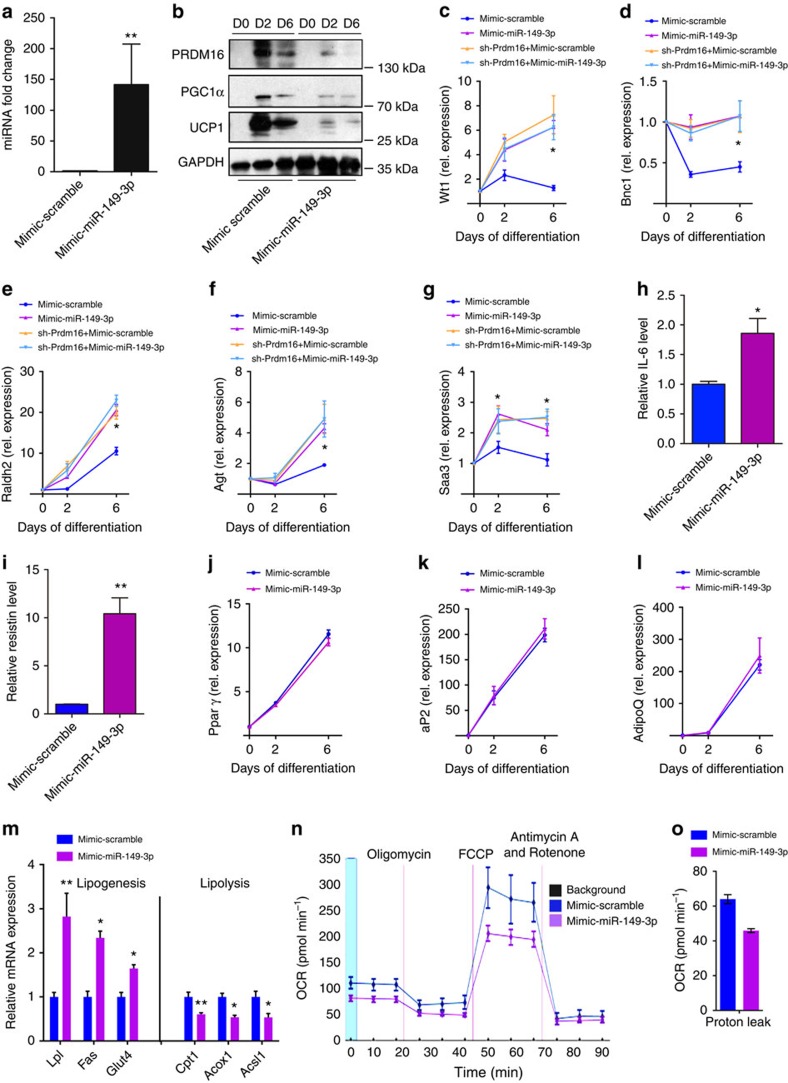
Overexpression of miR-149-3p induces visceral-selective gene expression in differentiated inguinal adipocytes. (**a**) Relative expression level of miR-149-3p in inguinal SV cells transfected with mimic-miR-149-3p. (**b**) Western blot analysis of PRDM16, PGC-1α and UCP1 levels in inguinal SV cells transfected with mimic-miR-149-3p or mimic-miR-control (mimic-scramble) at the indicated time points (day 0, day 2 and day 6) during inguinal SV cell differentiation). (**c**–**g**) Relative mRNA expression of *Wt1* (**c**), *Bnc1* (**d**), *Raldh2* (**e**), *Agt* (**f**), and *Saa3* (**g**) in inguinal SV cells infected with adenovirus expressing a shRNA targeted to Prdm16, co-transfected with mimic-miR-149-3p or mimic-miR-control during differentiation. (**h**,**i**) ELISA analysis of IL-6 (**h**) and Resistin (**i**) expression in inguinal SV cells in differentiation medium at day 6. (**j**–**l**) Relative mRNA expression of the adipogenic marker genes *Pparγ* (**j**), *aP2* (**k**) and *AdipoQ* (**l**), (**m**) Relative mRNA expression of lipogenesis and lipolysis genes in inguinal SV cells at day 6. The data show the mean of five independent experiments. (**n**,**o**) Oxygen consumption rates (OCRs) were quantified under basal conditions and with drugs that disrupt the respiratory chain using a Seahorse Biosciences XF 96 analyser in 6-day differentiated inguinal adipocytes transfected with mimic-miR-149-3p or mimic-miR-control. Experiments were performed in triplicated wells for each condition and repeated five times independently. The data represent the mean±s.e.m. *P<0.05; **P<0.001; (Student's *t*-test).

To further investigate the functions of miR-149-3p in inguinal adipocytes, we overexpressed miR-149-3p in inguinal preadipocytes (Fig. 6a). During differentiation, overexpression of miR-149-3p decreased the protein levels of PRDM16, PGC-1α and UCP1 compared with controls, in addition to repressing the brown fat-selective genes *Cox7a* and *Cox8b* (Fig. 6b; Supplementary Fig. 6a–c). In contrast, miR-149-3p overexpression markedly increased the visceral-selective genes, *Wt1*, *Bnc1 Raldh2, Agt* and *Saa3*, in differentiated cells, as well as the secreted proteins IL-6 and Resistin in cultured medium, compared with cells transfected with control-miR. However, in PRDM16-depleted cells, overexpression of miR-149-3p failed to induce visceral-selective inflammatory gene expression (Fig. 6c-i). Ectopic miR-149-3p expression did not influence the adipocyte differentiation *per se* (Fig. 6j–l; Supplementary Fig. 6d). Moreover, increased lipogenesis and decreased lipolysis were observed in cells overexpressing miR-149-3p (Fig. 6m). Importantly, overexpression of miR-149-3p led to a marked reduction in mitochondrial respiration, indicating a functional change in the differentiated inguinal adipocytes (Fig. 6n,o). Therefore, overexpression of miR-149-3p caused an impaired thermogenic programme along with the acquisition of partial visceral-selective characteristics during the course of inguinal adipocyte differentiation. However, neither of these alterations were observed in epididymal adipocytes overexpressing miR-149-3p (Supplementary Fig. 6f–n).

### ingWAT inhibition of miR-149-3p increases mice thermogenesis

**Figure 7:**
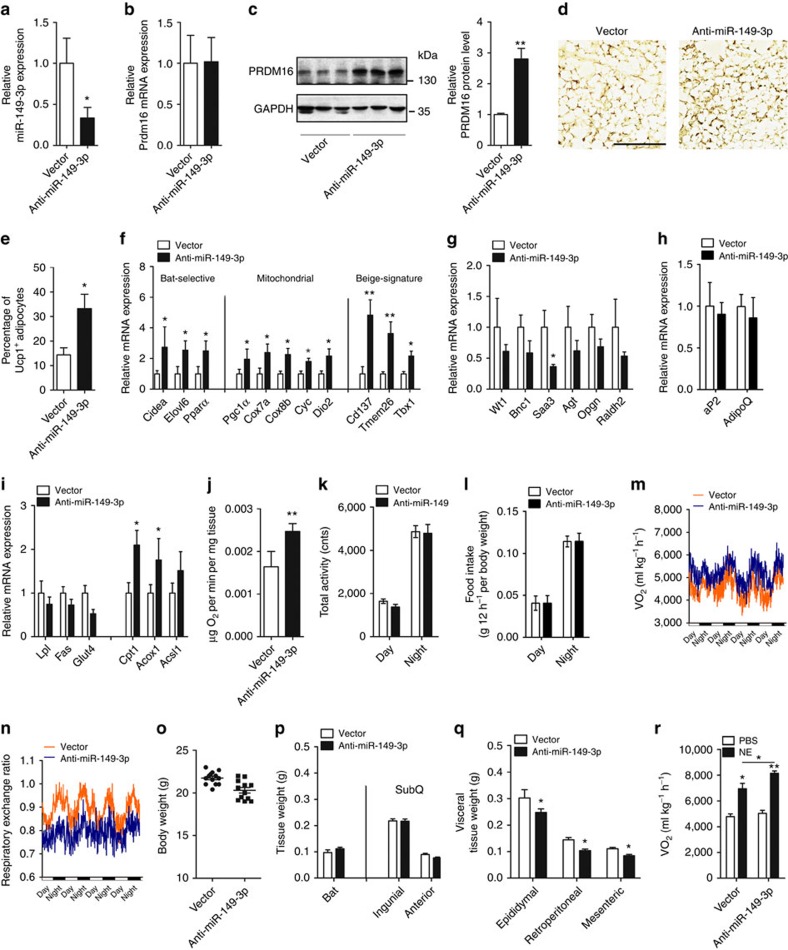
Subcutaneous inhibition of miR-149-3p induced browning of ingWAT in mice. (**a**–**r**) Lentiviral expression constructs containing scrambled control (vector) or antisense-miR-149-3p (anti-miR-149-3p) were used for inguinal adipose infection in male mice (*n*=8). (**a**) Relative expression level of miR-149-3p in inguinal adipose tissue infected with anti-miR-149-3p lentivirus vector (*n*=8). (**b**,**c**) Analysis of Prdm16 mRNA (*n*=8) (**b**) and protein (**c**) levels in inguinal adipose tissue infected with LV-vector or LV-antisense-miR-149-3p. GAPDH was used as an internal control. (**d**) Immunohistochemical staining for UCP1 abundance in respective inguinal sections. Scale bar, 100 μm. (**e**) Percentage of UCP1^+^ adipocytes in sections (*n*=5). (**f**–**i**) Normalized expression of BAT-selective genes, mitochondrial genes, beige-signature genes (**f**), visceral signature genes (**g**) adipose marker genes (**h**) and lipid metabolism genes (**i**) in inguinal adipose tissue in LV-vector or LV-antisense-miR-149-3p infected mice (*n*=8). (**j**) O_2_ consumption by inguinal adipose tissue (*n*=8). (**k**–**n**) Total activity (**k**), food intake (**l**), O_2_ consumption (**m**), and respiratory exchange ratio (**n**) in LV-vector or LV-antisense-miR-149-3p infected male mice (*n*=8). (**o**–**q**) Body weight (**o**), weight of brown fat mass, SubQ fat mass (**p**) and visceral fat mass (**q**) in LV-vector or LV-antisense-miR-149-3p infected mice (*n*=8). (**r**) O_2_ consumption in LV-vector or LV-antisense-miR-149-3p infected mice treated with NE or PBS (*n*=8). NE, Norepinephrine; SubQ, subcutaneous. The data represent the mean±s.e.m. *P<0.05; **P<0.001; (Student's *t*-test).

To identify the role of miR-149-3p in a purely *in vivo* context, a lentiviral vector expressing anti-miR-149-3p was directly introduced into the inguinal depot of mice^31^,^32^,^33^. Specifically, 10^7^ lentiviral transducing particles (TU)/mouse lentiviral vectors were inoculated into inguinal fat by multi-point subcutaneous injection (Supplementary Fig. 7a). According to the immunofluorescence microscopy analysis, ∼25% of inguinal cells expressed GFP 1 week post infection, and the infection rate stabilized at nearly 35% 2–3 weeks after infection (Supplementary Fig. 7b–d). Three weeks post-infection, lentivirus-driven expression of anti-miR-149-3p in mice efficiently decreased miR-149-3p expression in ingWAT (Fig. 7a; Supplementary Fig. 7e). Although Prdm16 mRNA was unchanged, the loss of miR-149-3p robustly elevated the level of PRDM16 protein in ingWAT (Fig. 7b,c; Supplementary Fig. 7f). The induction of PRDM16 protein was highly correlated with browning effects, as determined by the induction of UCP1 expression in the ingWAT of miR-149-3p-depleted mice (Fig. 7d,e). The broad sets of genes (BAT-selective, mitochondrial oxidation and beige-signature genes) associated with the thermogenic programme were also markedly increased by miR-149-3p inhibition, especially the beige-signature genes (Fig. 7f). Although the visceral-selective genes were repressed by miR-149-3p inhibition in ingWAT, the expression levels of the general adipogenic markers *aP2* and *AdipoQ* were not affected (Fig. 7g,h). Furthermore, inhibition of miR-149-3p resulted in slightly decreased lipogenesis and markedly increased lipolysis, along with induced O_2_ consumption, suggesting that loss of miR-149-3p elevated energy expenditure in the ingWAT of mice (Fig. 7i,j). Considering striking effect of miR-149-3p deficiency in ingWAT, the mice were subjected to metabolic analysis. Physical activity and food intake were similar in both groups of mice (Fig. 7k,l). Importantly, inhibition of miR-149-3p in ingWAT increased O_2_ consumption and decreased RER, indicating a substantial elevation of fat-based fuel (Fig. 7m,n). Thus, the weights of six adipose depots were measured, including BAT; visceral WAT depots: RP, mes and epiWAT; and subcutaneou WAT depots: ing, and antWAT. The decreased visceral WAT appeared to account for the slight body decrease in weight in anti-miR-149-3p treated mice (Fig. 7o–q). The action of PRDM16 can be enhanced by cAMP treatment, which mimics adrenergic input. In our animal model, the level of PRDM16 protein in ingWAT was robustly enhanced by miR-149-3p inhibition. Thus, we treated both groups of mice with norepinephrine (NE), a selective β-adrenergic agonist. As expected, although the NE treatment increased O_2_ consumption in control mice, the energy expenditure induction was significantly enhanced in miR-149-3p-depleted mice (Fig. 7r). These results suggest that inhibition of miR-149-3p stimulates the thermogenic programme of ingWAT, leading to increased energy expenditure in mice.

### miR-149-3p causes partial visceralization of ingWAT in mice

**Figure 8:**
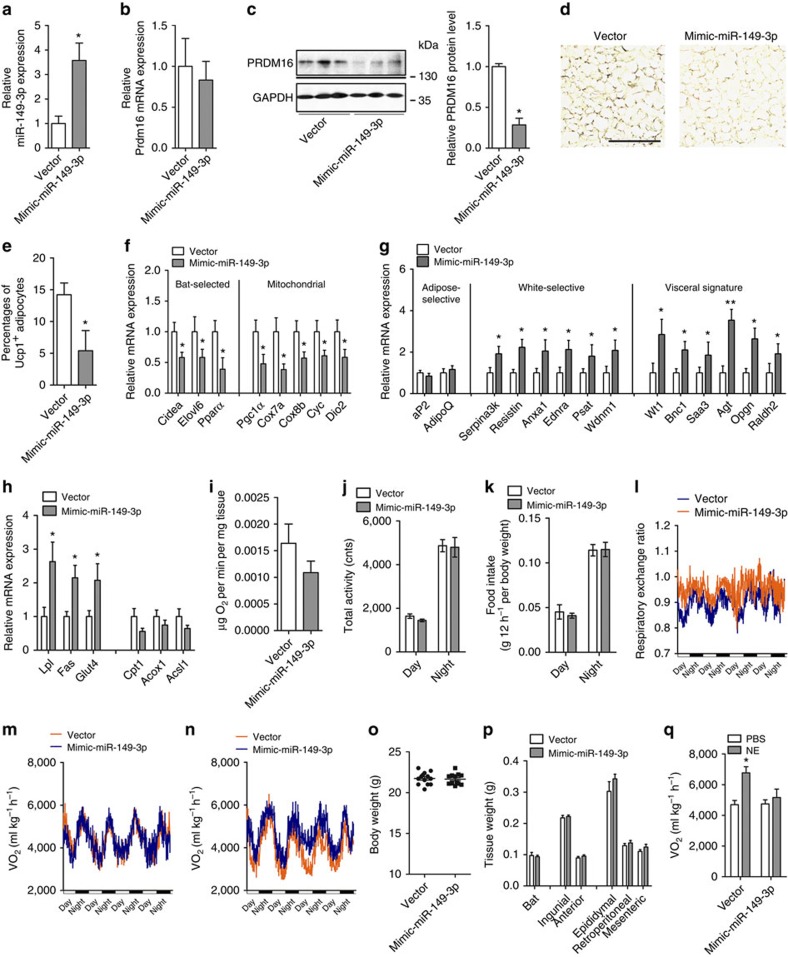
Subcutaneous overexpression of miR-149-3p induces a visceral-like phenotype in mouse inguinal adipose. (**a**–**q**) Lentiviral expression constructs containing scrambled control (vector) or mimic-miR-149-3p (mimic-miR-149-3p) were used for inguinal adipose infection in 6–8 week-old male mice (*n*=8). (**a**) Relative expression level of miR-149-3p in inguinal adipose tissue infected with mimic-miR-149-3p lentivirus vector (*n*=8). (**b**,**c**) Analysis of Prdm16 mRNA (**b**) and protein (**c**) levels in inguinal adipose tissue infected with LV-vector or LV-mimic-miR-149-3p. GAPDH served as an internal control. (**d**) Immunohistochemical staining for UCP1 abundance in respective inguinal sections. Scale bar, 100 μm. (**e**) Percentage of UCP1^+^ adipocytes in sections (*n*=5). (**f**–**h**) Normalized expression of BAT-selective genes, mitochondrial genes (**f**), adipose marker genes, white adipose selective genes, visceral signature genes (**g**) and lipid metabolism genes (**h**) in inguinal adipose tissue in LV-vector or LV-mimic-miR-149-3p infected mice (*n*=8). (**i**) O_2_ consumption by inguinal adipose tissue (*n*=8). (**j**–**n**) Total activity (**j**), food intake (**k**), respiratory exchange ratio (**l**), O_2_ consumption (**m**) and CO_2_ production (**n**) in LV-vector or LV-mimic-miR-149-3p infected male mice (*n*=8). (**o**,**p**) Body weight (**o**), weight of brown fat mass, SubQ fat mass and visceral fat mass (**p**) in LV-vector or LV-mimic-miR-149-3p infected mice (*n*=8). (**q**) O_2_ consumption in LV-vector or LV-mimic-miR-149-3p infected mice treated with NE or PBS (*n*=6). BAT, brown adipose tissue; NE, Norepinephrine. The data represent the mean±s.e.m. *P<0.05; **P<0.001; (Student's *t*-test).

Next, overexpression of miR-149-3p by lentivirus efficiently reduced the PRDM16 protein level in the ingWAT of mice, although only a downward trend in *Prdm16* mRNA expression was observed (Fig. 8a-c; Supplementary Fig. 7g). The ingWAT of mice overexpressing miR-149-3p showed reduced UCP1^+^ adipocytes along with reduced expression of a broad panel of thermogenic genes, including BAT-selective and mitochondrial genes (Fig. 8d-f). Although adipogenesis per se (*aP2* and *AdipoQ*) was not affected by ectopic miR-149-3p expression, the sets of classic WAT and visceral-selective genes were significantly increased (Fig. 8g). Given that miR-149-3p overexpression appeared to ‘visceralize' ingWAT at the molecular level, we assessed the physiological effects of this overexpression. In addition to increasing lipogenesis, ectopic miR-149-3p expression significantly reduced O_2_ consumption in ingWAT, suggesting visceral functional characteristics (Fig. 8h,i). Metabolic analysis showed no difference in food intake or activity between the two groups of mice. However, mice overexpressing miR-149-3p exhibited a markedly increased RER, suggesting a decrease in the utilization of fatty acid oxidation as an energy substrate (Fig. 8j–n). Although the overexpression of miR-149-3p in ingWAT resulted in a slight increase in visceral WAT, no significant alteration in whole-body weight was observed (Fig. 8o,p). We also studied these animals after injection with NE. Control animals showed a marked increase in O_2_ consumption following NE injection; however, overexpression of miR-149-3p in ingWAT blunted this NE-induced elevation, suggesting that this ingWAT-specific overexpression of miR-149-3p can affect whole-body energy expenditure (Fig. 8q).

## Discussion

The obesity epidemic has generated considerable interest in adipose tissue. The clinical description of obesity has largely been based on measurements that gauge total body fat^34^. However, scientists have recognized that the location of fat appears to have a close association with obesity. Visceral adiposity, which is more commonly observed among men than premenopausal women, is strongly associated with increased mortality^35^. However, the accumulation of subcutaneous adiposity has been termed ‘metabolically healthy obesity', which suggests that the distinct metabolic effects of visceral and subcutaneous WAT are most likely cell autonomous^36^. Fasting has been practiced for millennia and has been used as a powerful tool for studying the regulation of intermediary metabolism. Here, we showed that 24-h fasting triggered a depot-specific pattern of changes in both lipogenic and lipolytic genes in mice, indicating preferential mobilization of lipids in visceral depots compared with subcutaneous fat pads (Fig. 1). Food deprivation also stimulated a visceral-like switch in subcutaneous depots, from the morphological to the functional level (Figs 2 and 3). This observation may be evolutionarily important. During fasting, preferentially oxidized visceral fat can drain directly into the portal circulation and appears to be more efficient at meeting energy needs compared with the relatively externally located subcutaneous fat. Simultaneously, because large amounts of visceral fat are being used, subcutaneous fat must undergo a morphological and functional visceral-like switch to prepare to become a backup energy reservoir. Thus, under certain physiological circumstances, subcutaneous fat can be used to supplement the functions of visceral fat.

**Figure 9:**
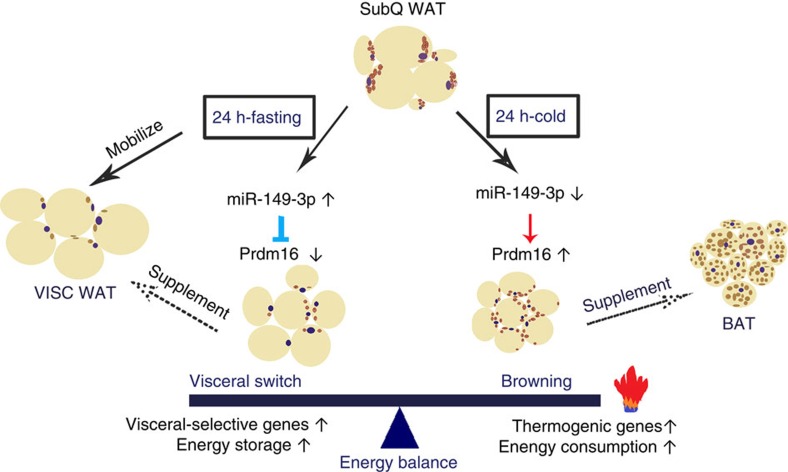
Subcutaneous WAT links energy balance through miR-149-3p-mediated regulation of Prdm16. After fasting for 24 h, subcutaneous ingWAT takes on many of the morphological and molecular characteristics of visceral fat to preserve energy via miR-149-3p-mediated suppression of PRDM16. By contrast, 24-h cold exposure decreased miR-149-3p and led to increased PRDM16 protein levels and adaptive thermogenesis in ingWAT. These findings unravel the extraordinary plasticity of subcutaneous WAT and its critical role in regulating energy homeostasis, especially in response to different physiological changes. BAT, brown adipose tissue; SubQ, subcutaneous; VISC, visceral; WAT, white adipose tissue.

Despite sharing the ability to accumulate triglycerides, the physiological roles of WAT and BAT are almost diametrically opposite^2^, which makes sense evolutionarily, because hunger and cold are two historical challenges during the development and evolution of mammals^15^. Although studies have demonstrated the existence of BAT in adult humans, it is still debated whether the amount of activated BAT in humans is sufficient to impact energy balance in a meaningful way^17^. However, subcutaneous WAT is very abundant in humans. Recent studies indicate that a subset of the precursor cells within subcutaneous adipose tissue can give rise to beige/brite cells, which are capable of defending against hypothermia and obesity^37^. However, beige cells are rarely observed in visceral fat. The striking but appreciated ‘browning' ability of subcutaneous has caused an explosion of interest in the function of this adipose tissue. Here, using a 24-h fasting stimulus, we found that fasting stimulated a set of visceral-selective gene transcripts but decreased the expression of genes related to the thermogenic programme (Fig. 3). This ‘whitening' of subcutaneous adipocytes intuitively makes sense, because it not only reduces heat production but also reserves energy to supplement visceral fat during fasting. Therefore, cold exposure and fasting, two different physiological stimuli, lead to nearly opposite phenotypic and functional changes in subcutaneous adipocytes to maintain the energy balance (Fig. 9). This extraordinary plasticity of subcutaneous adipocytes suggests that this adipose tissue might play even broader roles in the physiology and homeostasis of animals, particularly in humans.

miR-149-3p has rarely been investigated. Our study demonstrated that miR-149-3p directly targets and negatively regulates Prdm16 and that inhibition of miR-149-3p promotes the differentiation of precursors from subcutaneous to beige cells, thereby leading to increased mitochondrial activity (Figs 4 and 5). However, neither of these alterations was observed in miR-149-3p-depleted epididymal adipocytes, suggesting that miR-149-3p might have tissue-specific roles. This might be because the expression of Prdm16 is much lower in epiWAT than in ingWAT^1^. In addition, the manipulation of fat stores is an obvious therapeutic objective, but disruption of the normal differentiation or development of WAT causes lipodystrophy in both humans and experimental animals. Here, we demonstrated that subcutaneous inhibition by anti-miR-149-3p-activated beige cell development in ingWAT and subsequently increased whole-body energy expenditure without causing dysfunction in other tissues, which might be a potential strategy to counteract obesity (Fig. 7).

We are still in the process of understanding the similarities and differences between subcutaneous and visceral adipose tissue. Here, we show that in addition to the capability of ‘browning' to defend against hypothermia during cold exposure, subcutaneous WAT acquires many characteristics of visceral WAT to preserve energy during fasting via miRNA-mediated regulation of PRDM16. These data suggest an important role for subcutaneous in the regulation of energy homeostasis, especially when encountering different physiological changes.

## Methods

### Reagents and antibodies

FBS (cat# 16000-044), TRIzol reagent (cat# 15596-018), DMEM/F-12 (cat# 11330-032), and DMEM (cat# 11965-092) were purchased from Invitrogen (Carlsbad, CA, USA). SYBR-Green fluorescent dye (cat# 4368577) and TaqMan miRNA probes were purchased from Applied Biosystems (Foster City, CA, USA). Collagenase type II (cat# c6885), oligomycin (cat# 75351), FCCP (cat# C2920), rotenone (cat# R8875), indomethacin (cat# I-7378), dexamethasone (cat# D-1756), isobutylmethylxanthine (cat# I-5879), rosiglitazone (cat# R-2408), T3 (cat# T-2877) and the MystiCq microRNA qPCR Assay (cat# MIRRM02) were purchased from Sigma (Deisenhogfen, Germany).

For western blotting, anti-UCP1 antibody (cat# 14670) was purchased from Cell Signaling Technologies (Danvers, MA, USA), anti-PRDM16 antibody (cat# AF6295) was purchased from R&D Systems (Tustin, CA, USA)^21^, the anti-GAPDH antibody (cat# sc-25778) was purchased from Santa Cruz Biotechnology (Santa Cruz, CA, USA)^38^. For immunohistochemistry, anti-UCP1 antibody (cat# ab10983) was purchased from Abcam (Cambridge, MA, USA)^39^. The antibodies used for flow cytometry, including anti-CD45 (cat# 103121), CD11b (cat# 101207), and F4/80 (cat# 123115)^26^, were purchased from BioLegend (San Diego, CA, USA).

### Animals

All animal experimental procedures were conducted in accordance with the National Institutes of Health Guide for the Care and Use of Laboratory Animals and were approved by the Animal Care Committee of Nanjing University (Nanjing, China). Male or female C57BL/6J mice (6–8 weeks of age) were obtained from the Model Animal Research Center of Nanjing University and maintained on a standard diet (Research Diets cat# D10001, New Brunswick, NJ, USA) with a 12-h light cycle. For cold exposure, the mice were housed individually in a 4 °C incubator for 24 h with adequate food and water. For fasting, the mice were housed individually at 25 °C with water only. For fasting combined with cold exposure, the mice were housed individually in a 4 °C incubator for 24 h with water only. Experiments were performed with at least three independent cohorts.

### Cell culture

The stromal-vascular fractions of the inguinal and epididymal fat pads of 7–8-week-old male C57BL/6J mice were prepared and differentiated for 6 days as indicated in Supplementary Fig. 5a. The primary isolated preadipocytes were exposed to induction by DMEM/F-12 (Invitrogen cat# 11330-032, Carlsbad, CA, USA) containing indomethacin(125 μM; Sigma cat# I-7378), dexamethasone (5 μM; Sigma cat# D-1756), insulin (0.5 μg ml^−1^), isobutylmethylxanthine (0.5 mM; Sigma cat #I-5879), rosiglitazone (1 μM; Sigma cat# R-2408), T3 (1 nM; Sigma cat# T-2877), and 10% (vol/vol) FBS. From day 4 after induction, the cells were maintained in medium containing insulin (0.5 μg ml^−1^), T3 (1 nM), rosiglitazone (1 μM) and 10% (vol/vol) FBS until they were collected.

### Gene expression and western blotting

Total RNA from cultured cells or tissues was isolated using the TRIzol (cat# 15596-018) method (Invitrogen, Carlsbad, CA, USA). For mRNA quantitative PCR (qPCR) analysis, mRNA was reverse transcribed using the ABI high-capacity cDNA synthesis kit and was then used for quantitative PCR reactions with SYBR-Green fluorescent dye (ABI cat# 4368577, Foster City, CA, USA). The relative mRNA expression was determined after normalization to *Gapdh* levels using the ΔΔ*Ct* method. For western blot analysis, cells or tissues were lysed in RIPA buffer (0.5% Nonidet P-40, 0.1% sodium deoxycholate, 150 mM NaCl and 50 mM Tris-Cl at pH 7.5). Lysates were resolved by SDS–PAGE, transferred to a PVDF membrane (Millipore, Temecula, CA, USA), and probed with the indicated antibody. The anti-UCP1 (1:2,000) antibody (cat# 14670) was purchased from Cell Signaling Technology (Danvers, MA, USA). The anti-PRDM16 (1 μg ml^−1^) antibody (cat# AF6295) was purchased from R&D Systems (Tustin, CA, USA). The anti-GAPDH (1:1,000) antibody (cat# sc-25778) was purchased from Santa Cruz Biotechnology (Santa Cruz, CA, USA) and served as a loading control.

### miRNA expression analysis

For the microarray analysis, independent pooled inguinal adipose tissue samples were analysed from control, 24-h fasted and 24-h cold-exposed male C57BL/6J mice. Each sample comprised a pool of inguinal adipose tissues from four animals. Total RNA from each pooled sample was isolated using the TRIzol method for Affymetrix miRNA microarray analysis (CapitalBio Corp., Beijing, China). Procedures were performed as described on the web site of CapitalBio (http://www.capitalbio.com). Briefly, 50–100 μg of total RNA was used to extract miRNA with a miRNA Isolation Kit (Ambion Inc., Texas, USA). Biotin-labelled miRNAs were used for hybridization on each miRNA microarray chip containing probes in triplicate. Raw data were normalized to U6 and analysed using GenePix Pro 4.0 software (Axon Instruments, PA, USA). The following criteria were used to screen the miRNAs from the array data set: miRNAs with signal intensity greater than 30 were selected to avoid weak signal data; miRNAs from the 24-h fasting or 24-h cold exposure groups were each compared with those from the control group; after normalization, miRNAs that showed opposite expression ratios in the fasting and cold exposure groups were selected. The data were presented as a heat map with colour indicating the foldchange for each miRNA. Quantitative real-time PCR analysis was used to verify miRNA-149-3p expression. qPCR was performed using the MystiCq microRNA qPCR Assay (Sigma cat# MIRRM02, Deisenhogfen, Germany). TaqMan miRNA probes (Applied Biosystems, Foster City, CA, USA) were used to quantify the reported miRNA expressions levels, including miR-133a, the miR-365/193b cluster, and U6 snRNA, which was used as an internal control.

### Respiration

Tissue respiration was assessed using a Clark electrode (Strathkelvin Instruments, North Lanarkshire, Scotland). Fresh tissues were isolated from mice that were untreated, fasted for 24 h or exposed to cold for 24 h. The tissues were minced and placed in respiration medium (DPBS, 2 mm glucose, 1 mm pyruvate, 2% bovine serum albumin). O_2_ consumption was normalized to tissue weight. For each adipose depot, readings were taken using three separate pieces of tissue of equivalent size. Experiments were repeated five times independently.

### Flow cytometry

Epididymal visceral and inguinal subcutaneous adipose tissue were excised and digested using collagenase type II (cat# c6885; Sigma, Germany). Cell suspensions were filtered through a 40 mm sieve, and the SVF was collected after centrifugation at 450*g* for 10 min. To measure the cell sizes of the indicated adipocyte tissues, 2 × 10^4^ sample cells were analysed each time. To quantify macrophages, cells stained with anti-CD45 (cat# 103121), CD11b (cat# 101207), and F4/80 (cat# 123115) were purchased from BioLegend (San Diego, CA, USA). The cells were analysed using an LSRII instrument (BD Bioscience, New Jersey, USA) and FlowJo software (Single cell analysis, version 7.6.1, Ashland, Oregon).

### IHC and H&E staining

Tissues were fixed in 10% formalin, processed and embedded in paraffin. Multiple sections (10 μm in thickness) were prepared and stained with haematoxylin and eosin for morphological observation. For immunocytochemical staining, sections of adipose tissue were incubated with anti-UCP1 antibody (cat# ab10983; 1:1,000; Abcam, Cambridge, MA, USA) overnight at 4 °C. The signals were detected using a biotinylated goat anti-rabbit secondary antibody (cat# ba-1000; 1:1,000; Vector Laboratories, Burlingame, CA, USA) with the ABC kit (cat#PK-4001; Vector Laboratories, Burlingame, CA, USA) and DAB substrate (cat# h-2200; Vector Laboratories, Burlingame, CA, USA).

### Adenoviral infection and microRNA transfection

SV cell cultures at 70% confluence were incubated with adenovirus (MOI 50) expressing sh-Prdm16 or scrambled shRNA overnight in growth medium. The medium was then replaced, and cells were maintained in growth medium for an additional 36 h before miRNA transfection. Next, SV cells were trypsinized, collected by centrifugation, washed twice with PBS, and resuspended in DMEM/F-12. Transfections were performed at a concentration of 20 nM for the mimic- or anti-miRs, using the Gene Pulser Xcell Electroporation System (Bio-Rad, Hercules, CA, USA). The cells were then seeded in 6-well plates. After 4 h, the transfection complex was replaced with fresh adipogenic induction medium. After 2 days of induction, the medium was replaced with adipogenic maintainance medium and the cells were collected for RNA analysis after an additional 4 days of differentiation. All experiments were performed in triplicate wells for each condition and repeated five times independently.

### Luciferase assay

Plasmids carrying the Renila luciferase gene linked to a fragment of the Prdm16 3′UTR harbouring miR-149-3p putative binding sites were co-transfected into HEK293T cells (Human Embryonic Kidney, purchased from the Type Culture Collection of the Chinese Academy of Sciences, Shanghai, China, authenticated by STR Profiling, no mycoplasma contamination) along with control miRNA or miR-149-3p mimic (Genepharm, Suzhou, China). A mutant 3′-UTR of Prdm16 was constructed by mutagenesis of miR-149-3p from AGGGAGG into GGAGGGA. HEK 293T cells were cultured in DMEM (Gibco, Carlsbad, CA, USA) containing 10% FBS and seeded in 12-well plates. At 24 h after plating, 0.2 μg of firefly luciferase reporter plasmid, 0.2 μg of β-galactosidase (cat# 10586-014) expression vector (Ambion, Carlsbad, CA, USA), and equal amounts (20 pmol) of miR-149-3p mimic or scrambled negative control RNA were transfected into cells with Lipofectamine 2000 (cat# 11668-019) (Invitrogen, Carlsbad, CA, USA) according to manufacturer's instructions. A β-galactosidase vector was used as a transfection control. At 24 h post-transfection, the cells were analysed using a luciferase assay kit (cat# E4550) (Promega, Madison, WI, USA). All Experiments were performed in triplicate wells for each condition and repeated five times independently.

### Injection of lentiviruses in inguinal adipose tissue *in vivo*

The green fluorescent protein (GFP)-expressing HIV vector LV-pGLV-h-GFP-puro was purchased from GenePharma (Shanghai, China). Mouse miR-149-3p mimic and miR-149-4p antisense were packaged into lentiviruses by GenePharma. Six-week-old male mice were anaesthetized with 1% pentobarbital sodium; and anaesthesia was maintained during the surgical procedure. A short incision (∼5–8 mm) was made in the flank on both sides of the inguinal parts of the mouse, using a 30-gauge needle. Then, 50 μl of lentiviral particles (1 × 10^8^ lentiviral transducing particles (TU) ml^−1^, 100 μl per mouse) were administered directly by sight into the inguinal adipose tissues by 8–10 point injections on each side. Dispersion of the injected volume into the inguinal adipose tissue using this procedure was validated using a coloured dye in the preliminary experiments (Supplementary Fig. 7a). The incisions were then sutured, and the animals were housed at room temperature to recover. Immunofluorescence microscopy was used to visualize the infected cell and expressing GFP. The virus-infected mice were killed at the indicated time points post-infection (Supplementary Fig. 7b–d). Briefly, cryostat sections (8-μm thick) of the inguinal adipose tissues from mice 4, 7, 14 and 21 days post-infection were stained with DAPI and examined using an Olympus BX53 fluorescence microscope (Tokyo, Japan). As shown in Supplementary Fig. 7a, the multi-point injections were equally distributed among the adipose tissue; thus, five randomly selected visual fields/section of at least 10 nonsequential sections per mouse/time point were analysed (*n*=8). The percentage of GFP-positive areas were quantified by GFP signals/DAPI signals (Supplementary Fig. 7b). To quantify the percentage of infected cells expressing the constructs, the inguinal adipose tissues from mice 4, 7, 14 and 21 days post-infection were minced and, digested in PBS with collagenase for 60 min at 37 °C followed by dissociation. After centrifugation at 1,000 r.p.m. for 5 min, the total cells, including adipocytes (supernatant) and SV cells (bottom), were resuspended in 1 ml of PBS and mixed well. For observation in a single plane under the microscope, 15 μl of the cell mixture was placed between the slide and coverslip and eight random visual fields/slide were captured to determine the total cell number (bright field) and the number of GFP-positive cells. Each sample was measured 10 times, and the percentage of cells expressing GFP was calculated accordingly (Supplementary Fig. 7c,d). At 21 days post-infection, mice were either monitored in metabolic cages or euthanized for further experiments.

### Metabolic measurement

Experiments were conducted with 7-week-old male C57BL6/J mice, unless otherwise indicated. Energy expenditure was analysed using a Comprehensive LabMaster home cage system (TSE System, Thuringia, Germany). Following basal readings in the cages for 48 h, lentivirus-infected mice were injected subcutaneously with NE (1 mg kg^−1^).

### O_2_ consumption

Primary SV cells were cultured in 96-well plates and differentiated. Oxygen consumption rates (OCRs) were measured at basal glucose levels (Seahorse Bioscience, North Billerica, MA, USA), as well as with drugs disrupting the respiratory chain: oligomycin (ATP synthase inhibitor, 1 μM; Sigma-Aldrich cat#75351) and FCCP (uncoupler, 1 μM) (Sigma-Aldrich, cat# C2920). Finally, mitochondrial respiration was blocked with 1 μM rotenone (Sigma-Aldrich, cat# R8875). The residual OCR was considered non-mitochondrial respiration. All experiments were performed in triplicate wells for each condition and repeated five times independently.

### Statistics

The data were analysed using Prism 6 (GraphPad Software) with two-tailed Student's *t*-tests. For the energy expenditure results, the mean value was calculated for each mouse for each of the indicated durations, and the values were used to calculate the statistical significance between groups. Sample or experiment sizes were determined empirically to achieve sufficient statistical power. No statistical tests were used to predetermine the size of the experiments. *P* values from 0.001 to 0.05, or <0.001 were considered significant (*) or very significant (**), respectively. ‘NS' indicates no significance. All values are presented as the mean±s.e.m. unless otherwise indicated. In all of the experiments reported in this study, no data point were excluded. All data points are represented in the figures and were used in the statistical analyses. There was no blinding and no particular randomization method was used to assign individuals to experimental groups. Statistical analysis were performed using groups with similar variance. Limited variance was observed within sample groups.

## Additional information

**Accession codes:** Gene expression data have been deposited in GEO database under accession code GSE74753.

**How to cite this article:** Ding, H, *et al*. Fasting induces a subcutaneous-to-visceral fat switch mediated by microRNA-149-3p and suppression of PRDM16. *Nat. Commun.* 7:11533 doi: 10.1038/ncomms11533 (2016).

## Supplementary Material

Supplementary Information Supplementary Figures 1-7

## Figures and Tables

**Table 1:** Weight-related parameters of female mice.

	***Ad libitum***	**24 h of fasting**
Body weight at sacrifice (g)	16.830±0.307	13.330±0.494*
Food intake (g)	2.743±0.145	None
BAT (g)	0.120±0.003	0.110±0.008 (↓9% versus *ad libitum*)
SubQ ingWAT (g)	0.236±0.013	0.191±0.009* (↓19% versus *ad libitum*)
SubQ antWAT (g)	0.122±0.010	0.109±0.008* (↓11% versus *ad libitum*)
VISC POWAT (g)	0.206±0.023	0.096±0.019** (↓55% versus *ad libitum*)
VISC RPWAT (g)	0.050±0.003	0.022±0.003** (↓55% versus *ad libitum*)
VISC mesWAT(g)	0.131±0.010	0.052±0.004** (↓61% versus *ad libitum*)

ant, anterior; BAT, brown adipose tissue; ing, inguinal; mes, mesenteric; PO, periovarian; RP, -retroperitoneal; SubQ, subcutaneous; VISC, visceral.

Weight of brown adipose tissue (BAT) and subcutaneous inguinal, subcutaneous anterior, visceral periovarian and visceral retroperitoneal white adipose tissue (ingWAT, antWAT, POWAT and RPWAT, respectively) in C57BL/6J female mice fed ad libitum or fasted for 24 h. The percentage decrease in weight of fat depots after 24 h of fasting is indicated. The data represent the mean±s.e.m. *P<0.05; **P<0.001; (Student's *t*-test).
