# Supplementary material for: Fasting induces a subcutaneous-to-visceral fat switch mediated by microRNA-149-3p and suppression of PRDM16
Source: Nat Commun. 2016 May 31;7:11533. doi: 10.1038/ncomms11533 (PMC4895052; doi:10.1038/ncomms11533)
Supplement: Supplementary Information — Supplementary Figures 1-7 [file ncomms11533-s1.pdf]

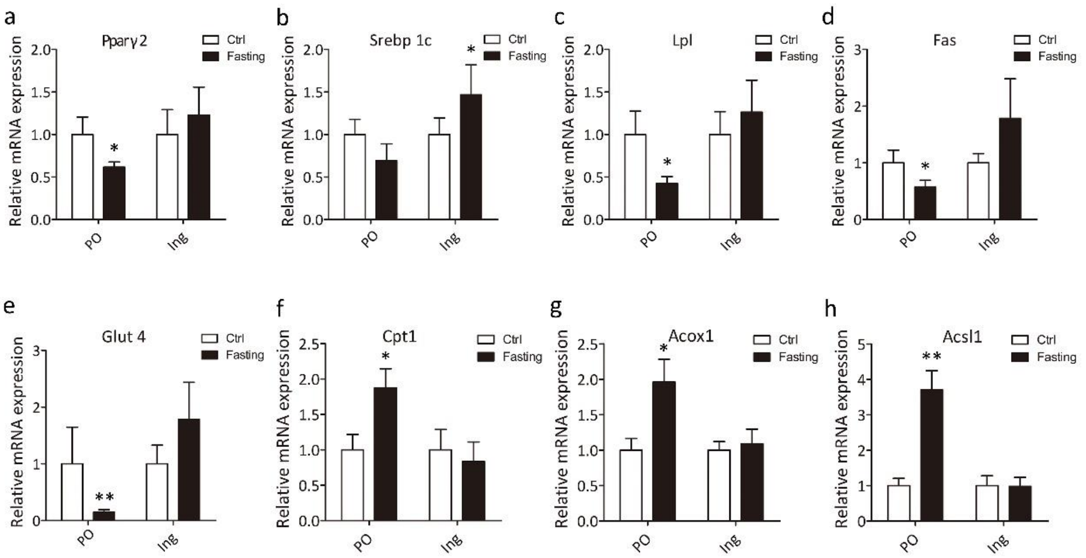

Supplementary Fig. 1. Fasting induces depot-specific mobilization of lipids in different adipose tissues in female mice**.** Expression of lipogenic and lipolytic genes in epididymal and inguinal depots from ad libitum fed or 24-h fasted female mice (n=8). Relative levels of lipogenic genes, *Pparγ2* (a), *Srebp1c* (b), *Lpl* (c), *Fas* (d), *Glut4* (e), and lipolytic genes, *Cpt1* (f), *Acox1* (g), *Acsl1* (h). PO, periovarian adipose tissue; Ing, inguinal adipose tissue. The data represent the mean ±s.e.m. *p < 0.05; **p <0.001 (Student's *t*-test).

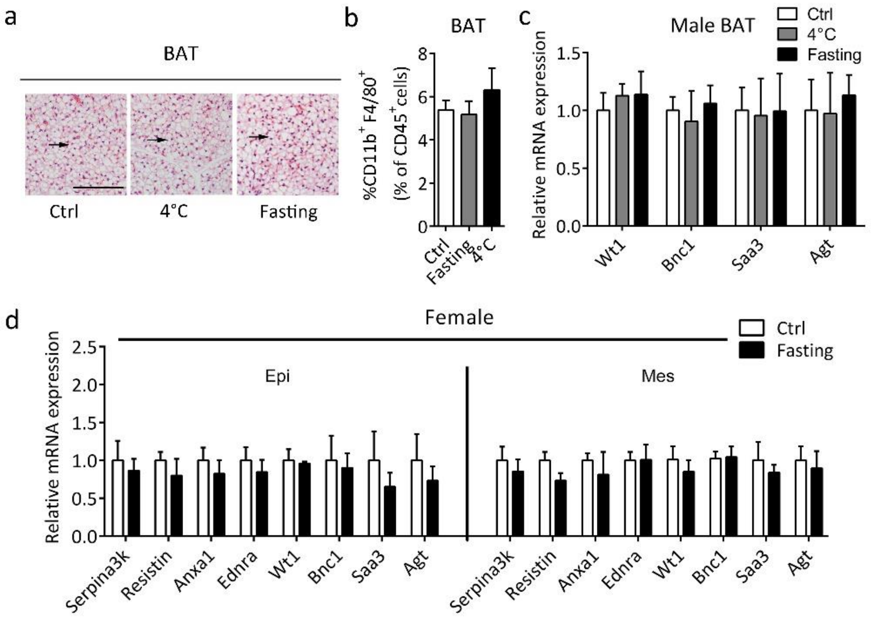

Supplementary Fig. 2. Morphology and gene expression of BAT in fasted mice and cold-exposed mice. (a) Representative images of haematoxylin and eosin (H&E) stained sections. Scale bar, 100 μm (n=8). (b) Flow cytometric quantitation of $CD11b^{+}F4/80^{+}$ macrophages in brown adipose tissue from ad libitum fed, 24-h fasted and 24-h cold exposed (4 ℃) male mice (n=6). (c) Normalized expression of visceral signature genes in brown adipose tissue from ad libitum fed, 24 h-fasted or 24 h-cold exposed (4 ℃) male mice (n=8). (d) Normalized expression of white-selective and visceral signature genes in epididymal and mesenteric adipose tissue from ad libitum fed or 24 h –fasted female mice (n=8). BAT, brown adipose tissue; Epi, epididymal adipose tissue; Mes, mesenteric adipose tissue. The data represent the mean ± s.e.m. (Student's *t*-test).

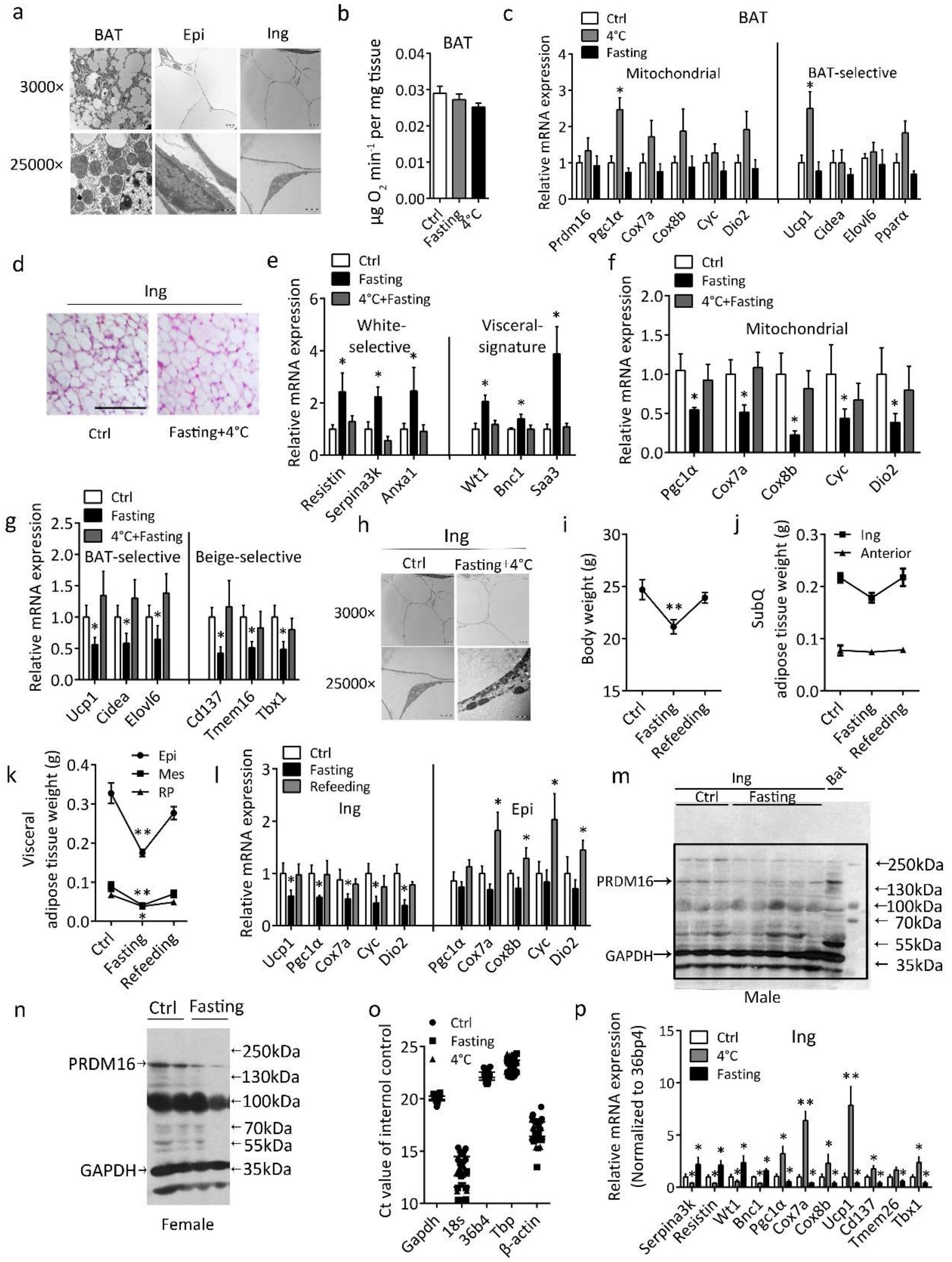

Supplementary Fig. 3. Morphology and gene expression of ingWAT from mice exposed to different physiological stimuli. (a) Transmission electron microscopy of brown, epididymal and inguinal adipose tissue. Scale bar, 5μm. (n=6). (b) O_2 consumption in brown adipose tissue from the three groups of mice (n=6). (c) Normalized expression of mitochondrial component and BAT-selective genes in brown adipose tissue in ad libitum, 24-h fasted, and 24-h cold

exposed male mice (n=8). (d-h) Mice were either fed ad libitum or fasted for 24 h at room temperature or 4 ℃. (d) Representative images of haematoxylin and eosin (H&E) stained sections (n=6). Scale bar, 100μm. (e-h) Normalized expression of white-selective genes, visceral signature genes (e), mitochondrial genes (f), BAT-selective, and beige-selective genes (g) in inguinal adipose tissue from ad libitum, 24-h fasted, and 24-h fasting combined with 4 ℃ exposed male mice (n=8). (h) Transmission electron microscopy of inguinal adipose tissue from ad libitum and 24h-fastting combined with cold exposure male mice. Scale bar, 5μm (n=5). (i-l) Effects of alternate-day fasting on different fat tissues. Male mice were fed ad libitum or refed for 24 h following a 24-h fast (n=8). Body weight (i), weight of SubQ fat mass (j) and visceral fat mass (k). (l) Normalized expression of mitochondrial component genes in inguinal and epididymal adipose tissue in the control, fasted and refed mice (n=8). (m) Full blots of Fig. 3j. (n) PRDM16 protein levels in ingWAT from female mice. Experiments were repeated three times independently. (o) Ct values for the internal control in inguinal adipose tissue from ad libitum fed, 24-h fasted or 24-h cold exposed mice (n=8). (p) Relative gene expression normalized to 36b4 from inguinal adipose tissue (n=8). BAT, brown adipose tissue; Ing, inguinal adipose tissue; Epi, epididymal adipose tissue; Mes, mesenteric adipose tissue; RP, retroperitoneal. The data represent the mean ± s.e.m. *p < 0.05; **p <0.001 (Student's *t*-test).

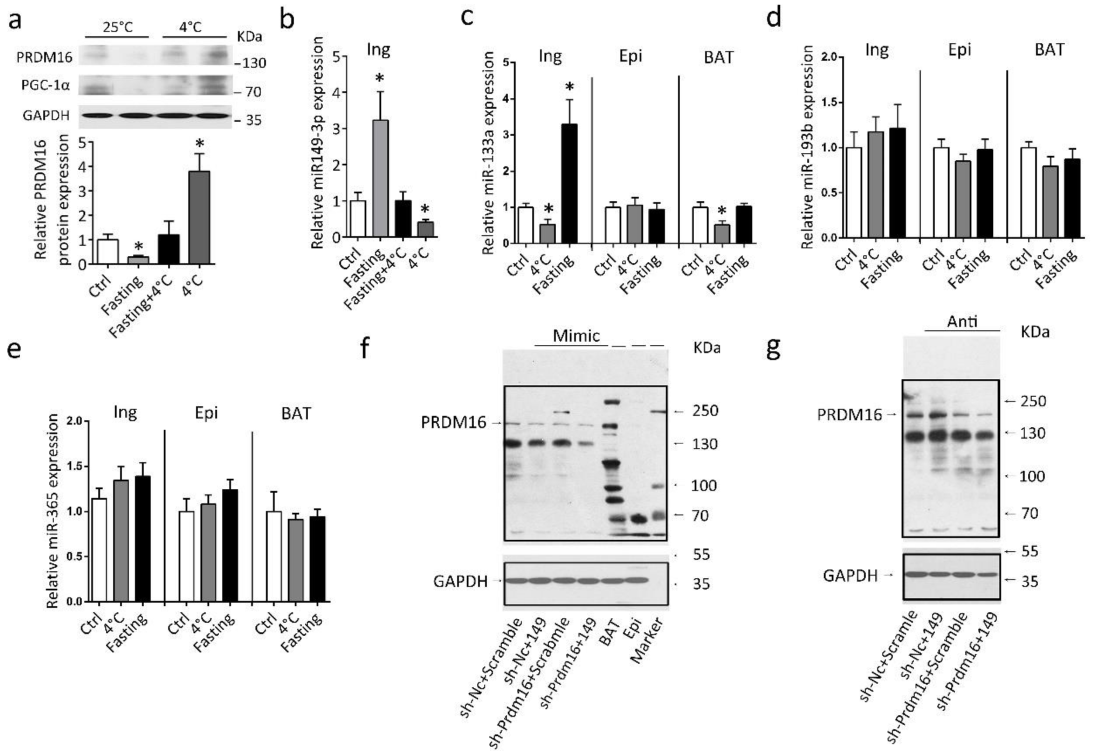

Supplementary Fig. 4. The miRNA expression levels in fasted and cold exposed mice. (a) Protein levels of PRDM16 and PGC-1α in inguinal adipose tissue from the 4 groups of mice (n=5). (b) Relative miR-149-3p expression in inguinal adipose tissue from ad libitum fed, 24-h fasted, 24-h cold exposed and 24-h fasted combined with 4 ℃ exposed male mice (n=8). (c-e) Relative miR-133a (c), miR-193b (d), and miR-365 (e) expression levels in inguinal, epididymal and brown adipose tissue from ad libitum fed, 24 h-fasted or 24 h-cold exposed (4 ℃) male mice (n=8). (f) Full blots of Fig. 4g. (g) Full blots of Fig. 4i. BAT, brown adipose tissue; Ing, inguinal adipose tissue; Epi, epididymal adipose tissue. The data represent the mean ± s.e.m. *$p < 0.05$ (Student's *t*-test).

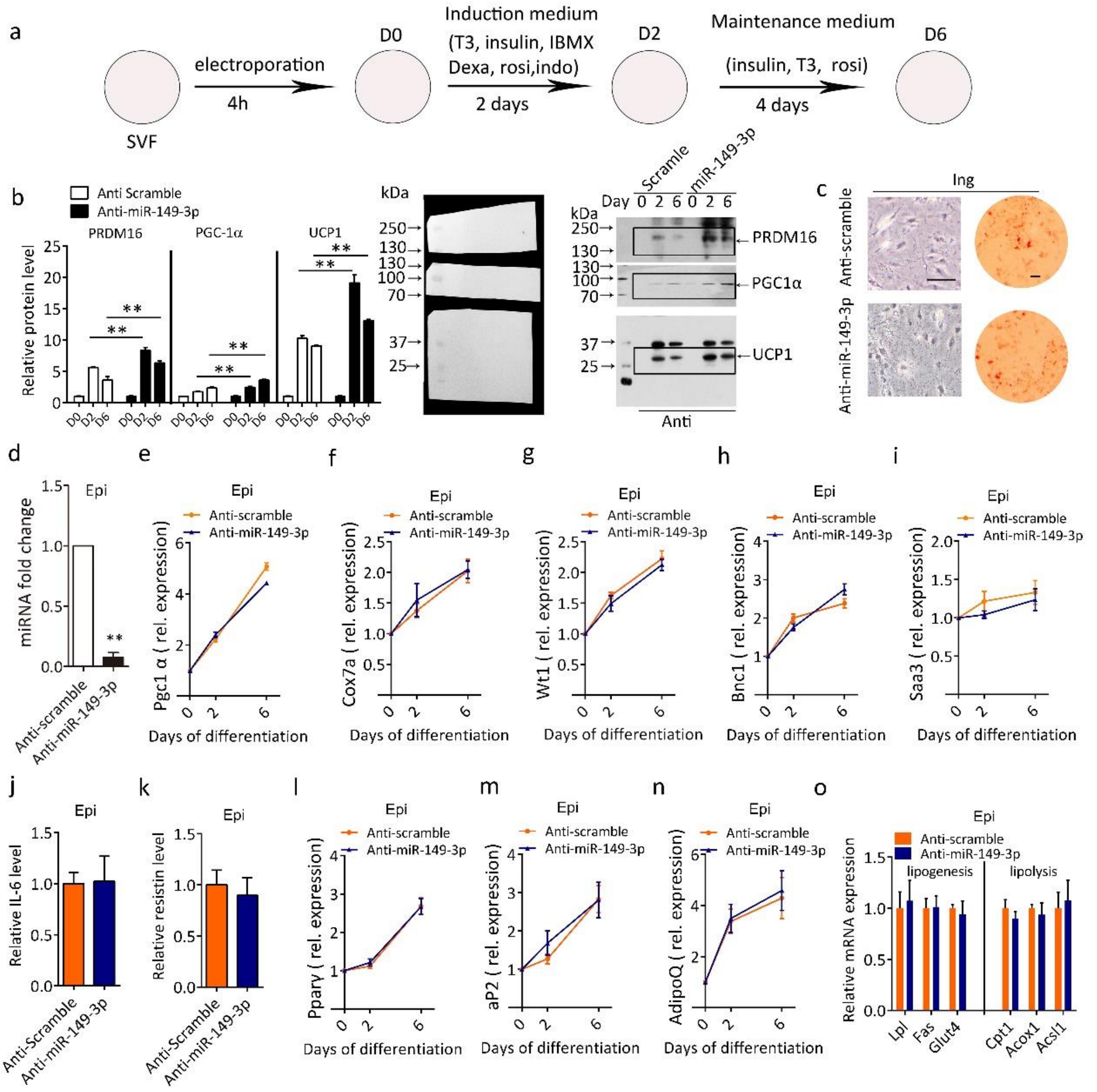

Supplementary Fig. 5. Depletion of miR-149-3p induces thermogenesis in differentiated inguinal adipocytes but not in epididymal adipocytes. (a) Schematic representation of the *in vitro* differentiation of SV adipocytes. (b) Densitometric analysis of PRDM16, PGC1α, and UCP1 protein levels and full blots of Fig. 5c. (n=5). (c) Morphology and oil red O staining of inguinal SVF transfected with anti-scramble or anti-miR-149-3p at day 6 of differentiation. Morphology, Scale bar, 100μm. Oil red O, Scale bar, 3mm (d) Relative expression level of miR-149-3p in epididymal SV cells transfected with anti-miR-149-3p (n=5). (e-i) Relative mRNA expression levels of *Pgc1α* (e), *Cox7a* (f), *Wt1* (g), *Bnc1* (h), and *Saa3* (i). (j, k) ELISA analysis of IL-6 (j) and Resistin (k) expression in epididymal SV cells in differentiation medium at day 6 (n=5). (l-n) Relative mRNA expression of *Pparγ* (l), *aP2* (m), and *AdipoQ* (n) in epididymal SV cells transfected with anti-miR-149-3p or anti-miR-control during

differentiation (n=5). (o) Relative mRNA expression of lipogenesis and lipolysis genes in epididymal SV cells at day 6 (n=5). All experiments were performed in triplicate wells for each condition and repeated five times independently. Ing, inguinal adipose tissue; Epi, epididymal adipose tissue. The data represent the mean ± s.e.m **p <0.001 (Student's *t*-test).

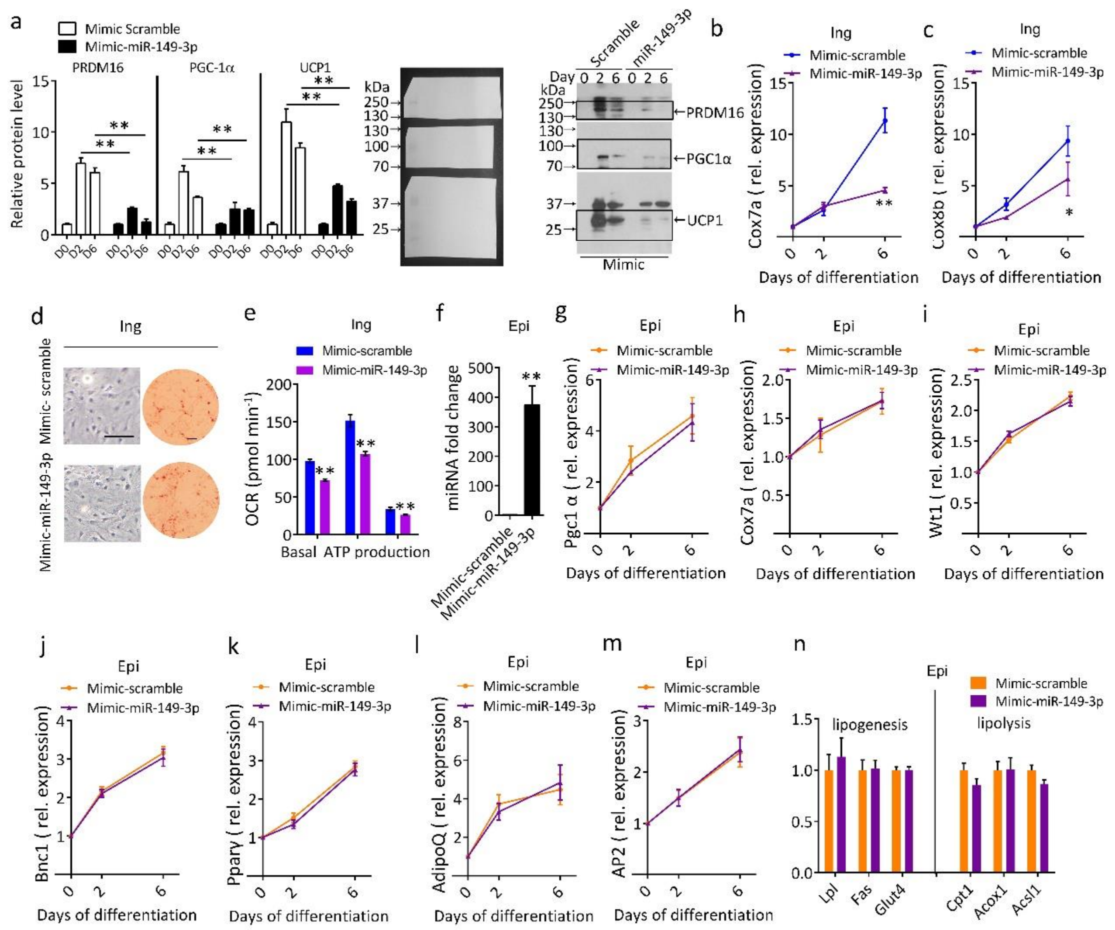

Supplementary Fig. 6. Ectopic miR-149-3p expression markedly reduces mitochondrial respiration in differentiated inguinal adipocytes but not in epididymal adipocytes. (a) Densitometric analysis of PRDM16, PGC1α, and UCP1 protein levels. (b, c) Relative mRNA expression of *Cox7a* (b) and *Cox8b* (c) in inguinal SV cells transfected with mimic-miR-149-3p or mimic-miR-control during differentiation (n=5). (d) Morphology and oil red O staining of inguinal SVF transfected with mimic scramble or miR-149-3p at day 6 of differentiation. Morphology, Scale bar, 100μm. Oil red O, Scale bar, 3mm. (e) Oxygen consumption rates (OCRs) were quantified at the basal level, as were spare respiratory capacity and ATP production in 6-day differentiated inguinal adipocytes transfected with mimic-miR-149-3p or mimic-miR-control (n=5). (f) Relative expression level of miR-149-3p in epididymal SV cells transfected with mimic-miR-149-3p (n=5). (g-m) Relative mRNA expression of *Pgc1α* (g), *Cox7a* (h), *Wt1* (i), *Bnc1* (j), *Pparγ* (k), *AdipoQ* (l), and *aP2* (m) in epididymal SV cells transfected with mimic-miR-149-3p or mimic-miR-control during differentiation (n=5). (n)

Relative mRNA expression of lipogenesis and lipolysis genes in epididymal SV cells at day 6 (n=5). Experiments were performed in triplicate wells for each condition and repeated five times independently. Ing, inguinal adipose tissue; Epi, epididymal adipose tissue. The data represent the mean ± s.e.m. **$p < 0.001$ (Student's *t*-test).

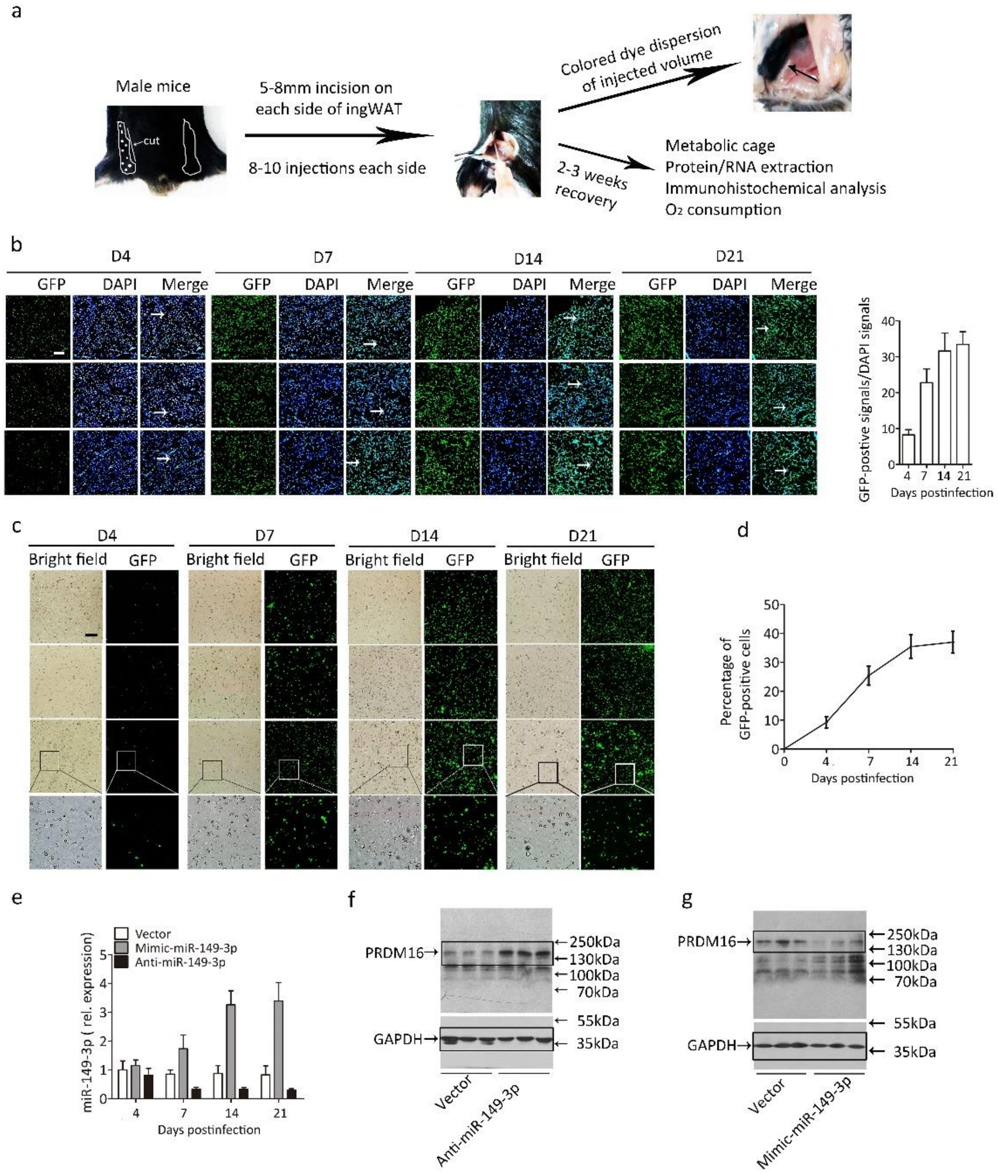

Supplementary Fig. 7. Lentiviral local delivery procedure and efficiency. (a) Schematic of the experimental procedure. (b-d) Infection efficiency of inguinal adipose tissues using the lentiviral vector (LV). (b) Green fluorescence protein (GFP) expression in cryostat sections of inguinal adipose tissue from LV-infected C57BL/6 mice were visualized by fluorescence microscopy (magnification, 10×) at different time points. Arrows indicate GFP expression (green) in adipocytes. Nuclei were counterstained with DAPI (blue). Scale bar: 100 μm. (c) Bright field and GFP photographs representative of cells from collagenase digested inguinal adipose tissue from LV-infected mice visualized by microscopy (magnification, 10× and 40×). Scale bar: 100 μm. (d) Quantification of the percentage of GFP^+ cells in inguinal adipose tissues from mice at day 4, day 7, day 14 and day 21 post-infection (n=8). (e) miR-149-3p expression

in LV-infected mice at the indicated time points (n=8). (f, g) Full blots of Fig. 7c and Fig. 8c. The data represent the mean ± s.e.m (Student's *t*-test).
